# *Fuyuanichthys wangi* gen. et sp. nov. from the Middle Triassic (Ladinian) of China highlights the early diversification of ginglymodian fishes

**DOI:** 10.7717/peerj.6054

**Published:** 2018-12-20

**Authors:** Guang-Hui Xu, Xin-Ying Ma, Yi Ren

**Affiliations:** 1 Key Laboratory of Vertebrate Evolution and Human Origins of Chinese Academy of Sciences, Institute of Vertebrate Paleontology and Paleoanthropology, Chinese Academy of Sciences, Beijing, China; 2 CAS Center for Excellence in Life and Paleoenvironment, Beijing, China; 3 Univesity of Chinese Academy of Sciences, Beijing, China

**Keywords:** Fossil, Ginglymodi, Holostei, Neopterygii, Actinopterygii

## Abstract

A series of well-preserved fossil assemblages from the Middle Triassic marine rock succession in Southwest China provide unique evidences for studying the early evolution of holostean fishes, including Halecomorphi (e.g., bownfin) and Ginglymodi (e.g., gars). Ginglymodi have the earliest record in the early Middle Triassic (Anisian, ∼244 Ma) of China, represented by *Kyphosichthys* and *Sangiorgioichthys sui* from Yunnan and *S. yangjuanensis* from Guizhou. Here, we report the discovery of a new ginglymodian, *Fuyuanichthys wangi* gen. et sp. nov., based on 22 well-preserved specimens from the lower part of the Zhuganpo member of the Falang Formation in eastern Yunnan and western Guizhou, which documents the first discovery of convincing ginglymodians from the late Middle Triassic (Ladinian, ∼240 Ma) Xingyi biota in China. *Fuyuanichthys* possesses a unique combination of features that easily distinguishes it from other ginglymodians, such as presence of a median gular and short and edentulous maxillae, and absence of a supramaxilla and supraorbitals. As one of the smallest known ginglymodians with a maximum standard length of ∼75 mm, the new finding further supports that the Middle Triassic Ginglymodi have a relatively small range of body sizes compared with the Halecomorphi from the same ecosystems in China. Results of a phylogenetic analysis recover *Fuyuanichthys* as a sister taxon to *Kyphosichthys* at the ginglymodian stem, and provide new insights into the early evolution of this clade.

## Introduction

Ginglymodi are a major lineage of ray-finned fishes, including gars and their closely-related fossil taxa (Regan, 1923; Patterson, 1973; Patterson, 1982; Grande, 2010; Cavin, 2010; López-Arbarello, 2012). They retain many primitive neopterygian features, e.g., thick dermal skull bones ornamented with enameloid tubercles, rhombic scales and fringing fulcra, and consequently are important for better understanding the early evolution of this clade. Living gars, represented by seven species in two genera of the family Lepisosteidae (Grande, 2010), are restricted to freshwater environments of North and Central America and Cuba, and all have an elongated body with long, toothed jaws, but extinct representatives of this group have a wider ecological adaptation and greater morphological diversity (Bartram, 1977; Schultze & Möller, 1986; Olsen & McCune, 1991; Maisey, 1991; Wenz, 1999; Bürgin, 2004; Cavin & Suteethorn, 2006; Tintori & Lombardo, 2007; Lombardo & Tintori, 2008; Xu & Wu, 2012; Schröder, López-Arbarello & Ebert, 2012; Cavin, Deesri & Suteethorn, 2013; Gibson, 2013; Deesri et al., 2014; López-Arbarello & Alvarado-Ortega, 2011; López-Arbarello et al., 2011; López-Arbarello et al., 2016; Brito, Alvarado-Ortega & Meunier, 2017). Recent studies based on morphological data (Grande, 2010; Deesri, Jintasakul & Cavin, 2016; López-Arbarello & Sferco, 2018), fitting well with molecular studies (Hurley et al., 2007; Near et al., 2012; Broughton et al., 2013; Betancur-R et al., 2017), consistently recover Ginglymodi as the sister group to Halecomorphi (*Amia* and fossil relatives; Olsen, 1984; Grande & Bemis, 1998).

**Figure 1:**
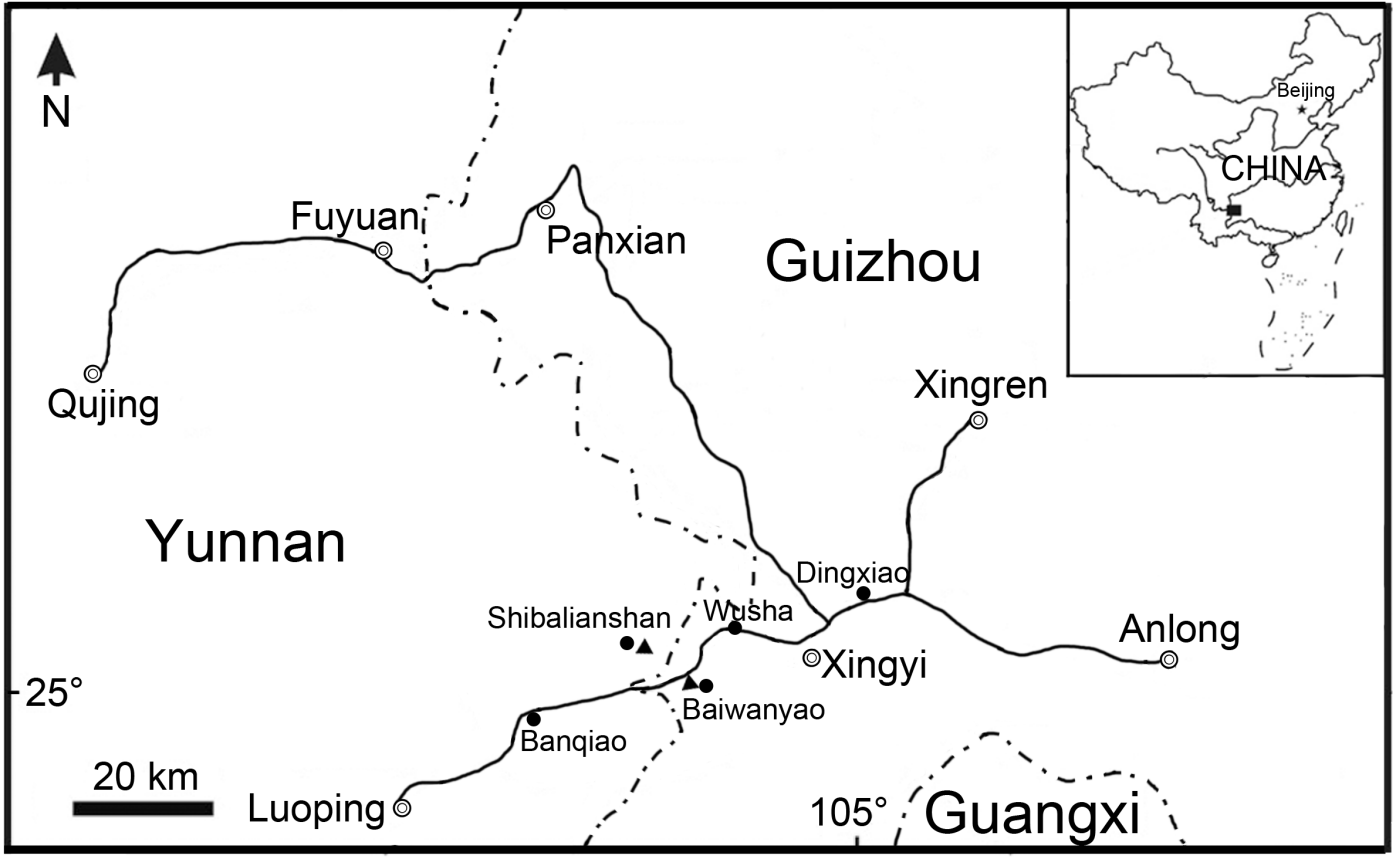
Fossil localities. Map showing the fossil localities of *Fuyuanichthys wangi* gen*.* et sp. nov.

Ginglymodi have the earliest fossil record in the early Middle Triassic (Anisian) of China (López-Arbarello & Sferco, 2018), represented by three ginglymodians from the Guanling Formation of Luoping, Yunnan Province (*Sangiorgioichthys sui*, López-Arbarello & Alvarado-Ortega, 2011; *Kyphosichthys*, Xu & Wu, 2012) and Panxian, Guizhou Province (*S. yangjuanensis*, Chen et al., 2014). Here, we report the discovery of a new ginglymodian based on 22 specimens from the lower part of the Zhuganpo member of the Falang Formation exposed in the Shibalianshan, Fuyuan, Yunnan and Baiwanyao, Xingyi, Guizhou, China (Fig. 1). Most specimens are nearly complete and well-preserved in the dark grey thin- to medium-bedded marlites or argillaceous limestones. Biostratigraphical studies (Chen, 1985; Li, 2006; Zou et al., 2015; Sun et al., 2016; Li et al., 2016a) generally suggest a late Middle Triassic (Ladinian) age for these fossil beds, as supported by the zircon U-Pb dating (240.8 ± 1.8 Ma; Li et al., 2016b). Also from the fossil beds are rich invertebrates, several other kinds of bony fishes, and diverse marine reptiles; the whole fossil assemblage represents the renowned Xingyi biota (Young, 1958; Su, 1959; Jin, 2001; Jin, 2006; Liu, Yin & Wang, 2002; Liu et al., 2003; Li, 2006; Geng, Zhu & Jin, 2009; Xu et al., 2012; Xu, Zhao & Shen, 2015; Xu, Ma & Zhao, 2018; Benton et al., 2013; Tintori et al., 2015; Xu & Ma, 2018). The discovery represents the first ginglymodian known in the Xingyi biota and provides new insights into the early evolution of this clade.

## Material and Methods

All specimens are curated at the fossil collections of the Institute of Vertebrate Paleontology and Paleoanthropology (IVPP), Chinese Academy of Sciences in Beijing, China. They were mechanically prepared with sharp steel needles. Illustrations were drawn manually and then prepared using Adobe Photoshop and Illustrator software packages (CS5). The relative position of fins and scale counts were expressed following Westoll (1944). The phylogenetic analysis was performed on the basis of a data matrix of 201 characters coded across 37 actinopteran taxa with *Pteronisculus stensioi* selected for out-group comparison (Supplemental Information). Besides the new taxon described here, the selected holosteans include 21 ginglymodians and eight halecomorphs. The taxonomically uncertain taxon *Luoxiongichthys* (Wen et al., 2012) was not included, pending its redescription and revision. *Sangiorgioichthys sui* (López-Arbarello et al., 2011) was not included either because it is currently being restudied based on new material. The characters were mainly adopted or modified from previous analyses of neopterygian phylogeny (Gardiner & Schaeffer, 1989; Gardiner, Maisey & Littlewood, 1996; Arratia, 1999; Arratia, 2013; Grande, 2010; Cavin, 2010; López-Arbarello, 2012; López-Arbarello et al., 2016; López-Arbarello & Sferco, 2018; Xu, Gao & Finarelli, 2014; Xu, Zhao & Coates, 2014; Xu, Gao & Coates, 2015; Xu & Zhao, 2016; Xu & Ma, 2016; Xu & Ma, 2018; Deesri, Jintasakul & Cavin, 2016; Giles et al., 2017; Sun & Ni, 2018). All characters were unordered and equally weighted. Tree searches were accomplished with the heuristic search algorithm (gaps treated as missing data; 10,000 random addition sequence replicates; tree bisection-reconnection (TBR) branch-swapping, with 10 trees held at each step and multiple trees saved) in PAUP* 4.0b10 (Swofford, 2003).

For ease of comparison with most existing literature, the traditional actinopterygian nomenclature of Grande & Bemis (1998) and Grande (2010) are generally followed. The segmented and unbranched ray just anterior to the branched rays is termed as the first principal ray in dorsal and anal fins. In caudal fin, the dorsal and ventral segmented and unbranched rays adjacent to the branched rays are termed as the first and last principal rays, respectively. All other segmented and unbranched rays are termed as rudimentary rays, following the nomenclature used in many other contributions (e.g., Alvarado-Ortega & Espinosa-Arrubarrena, 2008; López-Arbarello & Alvarado-Ortega, 2011), although the rudimentary rays are also termed as procurrent rays in the ventral lobe of the caudal fin by some authors (e.g., Arratia, 2013).

The electronic version of this article in Portable Document Format (PDF) will represent a published work according to the International Commission on Zoological Nomenclature (ICZN), and hence the new names contained in the electronic version are effectively published under that Code from the electronic edition alone. This published work and the nomenclatural acts it contains have been registered in ZooBank, the online registration system for the ICZN. The ZooBank LSIDs (Life Science Identifiers) can be resolved and the associated information viewed through any standard web browser by appending the LSID to the prefix http://zoobank.org/. The LSID for this publication is: urn:lsid:zoobank.org:pub:69D69D4F-1F36-472D-BBA5-BA37E22E360B. The online version of this work is archived and available from the following digital repositories: PeerJ, PubMed Central and CLOCKSS.

## Systematic Palaeontology

**Table:** 

Holostei Müller, 1844
Ginglymodi Cope, 1872
*Fuyuanichthys* gen. nov.

**Etymology**. The generic epithet ‘Fuyuan’ refers to Fuyuan County (Qujing, Yunnan), and the Greek suffix ‘ichthys’ means fish.

**Type species**. *Fuyuanichthys wangi* gen. et sp. nov.

**Diagnosis**. Same as for the type and only known species.

*Fuyuanichthys wangi* gen. et sp. nov.

**Etymology**. The specific epithet honors Mr. Kuan Wang, who contributed to the fossil collecting.

**Figure 2:**
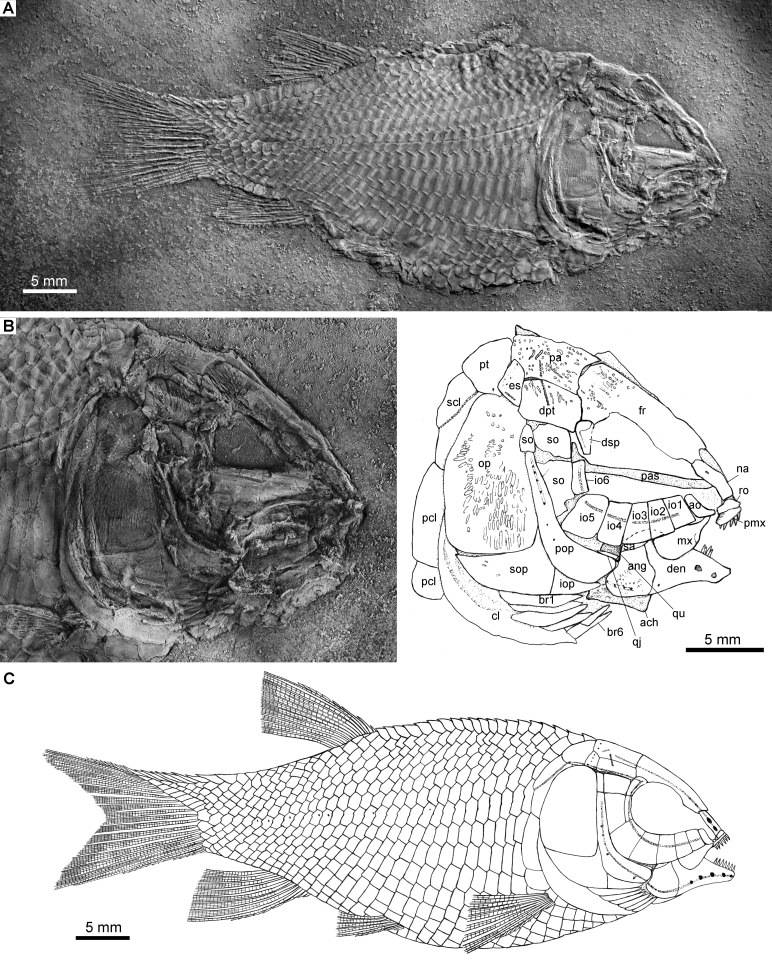
Holotype and reconstruction. *Fuyuanichthys wangi* gen*.* et sp. nov. (A) Complete skeleton and (B) Skull and pectoral girdle of IVPP V22923 (holotype) coated with ammonium chloride. (C) Reconstruction.

Holotype. IVPP V22923, a complete, laterally compressed specimen from Shibalianshan, Fuyuan, Yunnan (Fig. 2A).

**Referred Material**. IVPP V 19980, 19989–19993 from Baiwanyao, Xingyi, Guizhou; IVPP V 22920–22922, 22924–22926, 24266–24274 from Shibalianshan, Fuyuan, Yunnan.

**Locality and horizon**. Shibalianshan, Fuyuan, Yunnan, and Baiwanyao, Xingyi, Guizhou, China; lower part of Zhuganpo member of the Falang Formation, Ladinian (240.8 ± 1.8 Ma; Li et al., 2016b), Middle Triassic.

**Diagnosis**. A small-sized ginglymodian distinguished from other members of this clade by the following combination of features: body depth 42–46% of standard length; frontal 1.8–2.0 times as long as parietal; absence of supraorbitals; absence of supramaxilla; maxilla relatively short and edentulous, ending below anterior orbital margin; large sensory pores in nasal and dentary; L-shaped preopercle; six infraorbitals (including two anterior infraorbitals); two large suborbitals posterior to circumorbtial bones, and single small suborbital between preopercle and dermopterotic; six pairs of branchiostegal rays; presence of median gular; scales smooth with straight posterior margin; 9–11 dorsal rays; eight principal anal rays; 16–18 principal caudal rays; and scale formula of D20/P8–9, A14–15, C25–27/T29–31.

## Description

### Shape and size

*Fuyuanichthys* has a blunt snout, a fusiform body and an abbreviated heterocercal caudal fin with a forked profile. The holotype (Fig. 2) has a standard length (SL) of 49.1 and a head length of 18.4 mm; the largest specimen (IVPP V19992) has a SL up to 74.8 mm. The greatest body depth lies midway between the posterior margin of the opercle and the origin of the dorsal fin, and accounts for 42–46% of the SL (Table 1).

**Table 1:** Measurement data (in mm).

Specimens	SL	HL	BD	PVL	PDL	PAL	TL
V19980	72.3	26.9	32.6	43.9	51.5	58.8	–
V19989	68.1	24.5	32.3	–	51.2	54.0	82.6
V19990	46.7	20.1	21.6	30.9	34.7	38.9	60.8
V19992	74.8	27.9	35.3	44.4	52.3	59.3	99.3
V19993	62.1	24.1	31.6	–	47.5	50.7	83.3
V22920	38.9	14.9	17.3	23.5	27.3	30.1	48.6
V22922	55.5	21.6	28.2	36.6	40.8	45.6	69.8
V22923	49.1	18.4	22.6	30.8	35.2	40.1	63.6
V22924	63.3	24.5	30.2	40.0	47.4	50.5	86.5
V22925	55.0	21.4	23.0	37.3	40.3	46.3	72.7
V24268	50.0	21.1	22.8	31.8	36.3	41.2	62.1
V24271	54.7	19.8	24.3	34.4	38.7	45.8	72.2
V24273	56.5	22.6	24.8	37.3	40.7	46.2	73.0

**Notes.**

BD body depth HL head length PAL preanal length PDL predorsal length PVL prepelvic length SL standard length TL total length

### Snout

**Figure 3:**
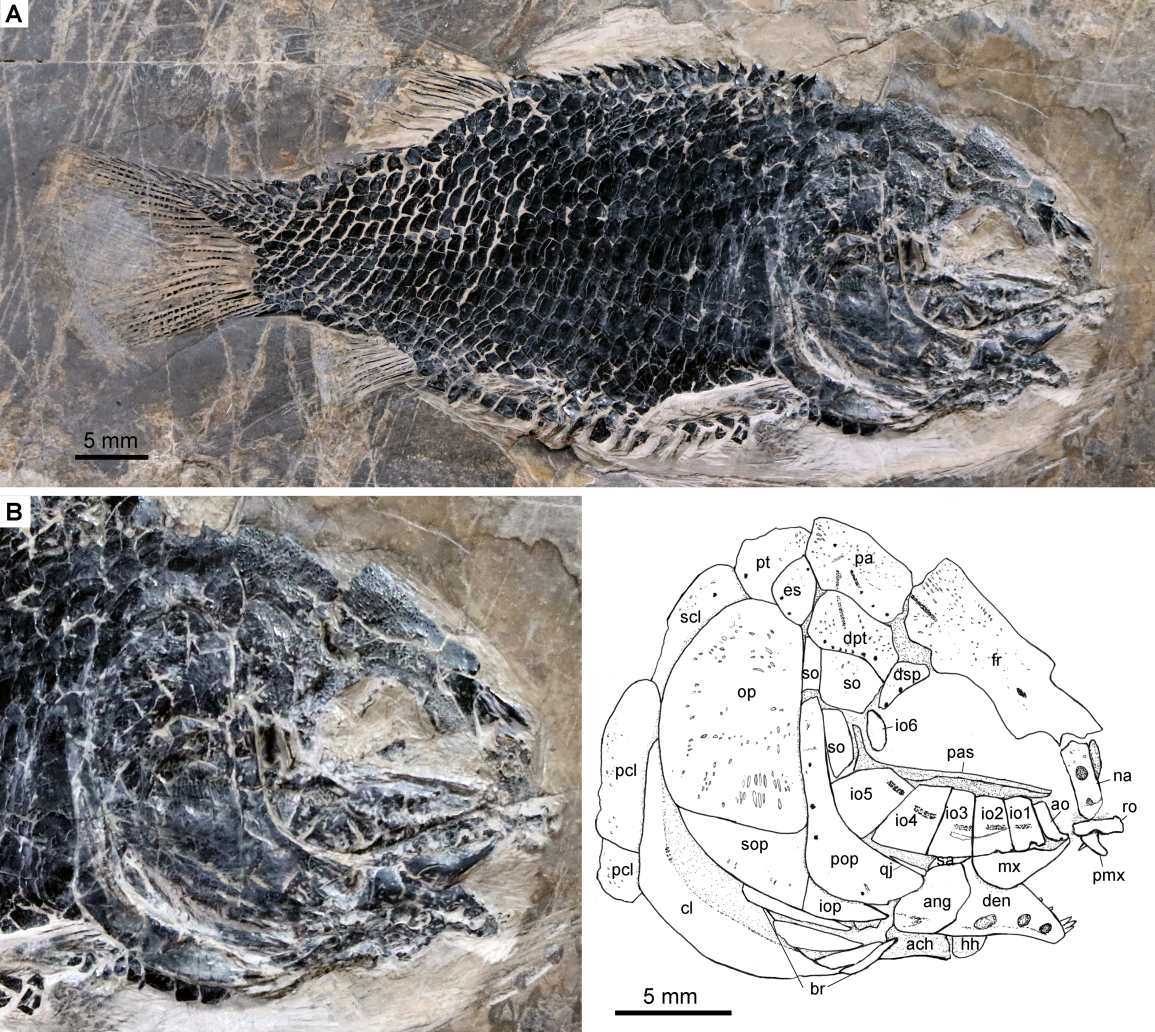
Specimen IVPP V24273. *Fuyuanichthys wangi***gen*.* et sp. nov., IVPP V24273.** (A) complete skeleton. (B) skull and pectoral girdle.

**Figure 4:**
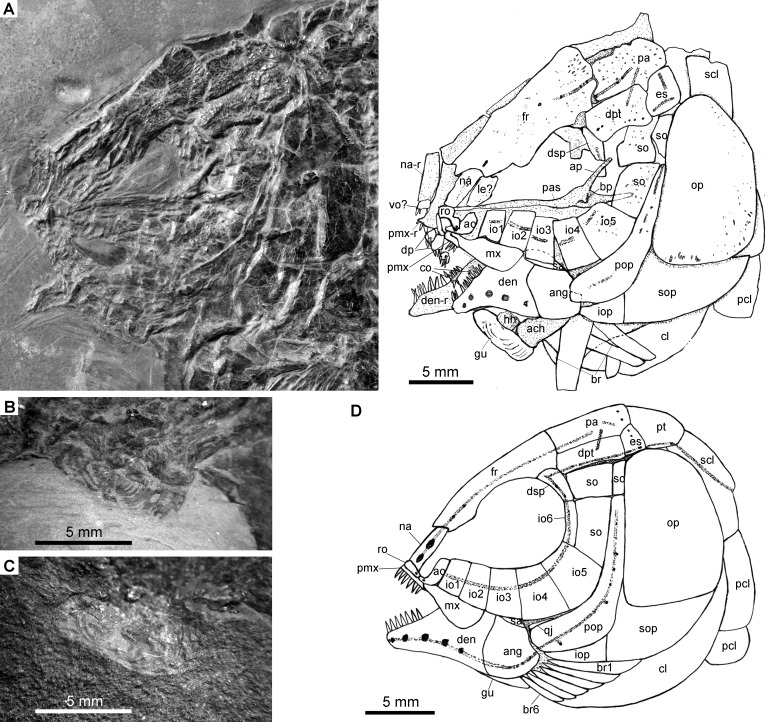
Specimens 19992-19993. *Fuyuanichthys wangi***gen*.* et sp. nov.** (A) Skull and pectoral girdle, IVPP V19992. (B) Close-up of median gular in IVPP V19992, immersed in water when photographed.** (C) Close-up of median gular, IVPP V19993. (D) Reconstruction of skull and pectoral girdle.

The canal-bearing bones in the snout region include a median rostral and a pair of nasals and antorbitals (Figs. 2–4). The median rostral is a small, dorsoventrally short bone with a concaved anterior margin and a slightly convex posterior margin (Fig. 4). It contacts the nasals posterodorsally and overlies the anterior portions of the premaxillae. The ethmoid commissure is housed in this bone. The nasal is elongate and rectangular, without obvious notches for nostrils. The pores for the supraorbital sensory canal in the nasal are notably large (Fig. 3B). A similar condition is otherwise known in *Kyphosichthys* (Xu & Wu, 2012; Sun & Ni, 2018). The antorbital is small and L-shaped, with a relatively short, canal-bearing anterior arm.

### Skull roof

The skull roofing bones include a pair of frontals, parietals, dermopterotic, and extrascapulars. The outer surfaces of these bones are ornamented with ganoine ridges and tubercles. The frontals are elongate, 1.8–2.0 times as long as the parietal (Figs. 2–4). Each frontal contacts both the parietal and dermopterotic with a convex posterior margin, and the nasal with a concave anterior margin. It gradually widens posteriorly, having a conspicuous constriction above the orbital region. The parietals are roughly rectangular and twice as long as its width, with a triangular posterolateral process inserting between the dermopterotic and extrascapular. There are three pit-lines on each parietal; the anterior pit-line originates at the middle portion of the parietal, extends anteriorly and nearly contacts the supraorbital sensory canal; the middle pit-line extends laterally into the middle portion of the dermopterotic; and the posterior pit-line is confined in the posterolateral portion of this bone.

The dermopterotics are roughly rectangular or somewhat hourglass-shaped with a constricted middle portion. The medial margin is nearly straight or slightly convex and the lateral margin slightly concave. Each dermopterotic contacts both the frontal and dermosphenotic anteriorly, and the extrascapular posteriorly. The temporal sensory canal runs longitudinally through the dermopterotic near the lateral margin of this bone.

A pair of extrascapulars is present. They are sub-triangular, tapering medially. The left is separated from contact with the right by the posterior portions of the parietals (Figs. 2– 4). The supratemporal commissural canal runs transversely through both extrascapulars and parietals. Similar conditions are otherwise known in *Sangiorgioichthys sui* (López-Arbarello et al., 2011), *Kyphosichthys* (Xu & Wu, 2012; Sun & Ni, 2018), *Macrosemimimus* (Schröder, López-Arbarello & Ebert, 2012) and macrosemiids (Bartram, 1977).

### Circumorbtial series

There are six rectangular or trapezoidal infraorbitals between the antorbital and the dermosphenotic (Figs. 2–4). Among them, the first two infraorbitals (=anterior infraorbitals; Wenz, 1999; López-Arbarello & Sferco, 2018) are positioned anterior to the orbital margin that can be inferred from the sclerotic ring. The third and fourth infraorbtials are 1.6–1.8 times deeper than long, forming the ventral margin of the orbit. The fifth contacts the preopercle posteriorly, being the largest element of the infraorbital series. The third to fifth infraorbitals are ventrally or posteroventrally expanded, resembling the conditions in several other Triassic ginglymodians, e.g., *Lophionotus sanjuanensis* (Gibson, 2013), *Kyphosichthys* (Xu & Wu, 2012; Sun & Ni, 2018), and *Semiolepis* (Lombardo & Tintori, 2008). The last is small, deep and narrow, forming a part of the posterior orbital margin.

The dermosphenotic is deep and trapezoidal, contacting the dermopterotic and suborbital posteriorly, the frontal dorsally, and the last infraorbital ventrally. Two or three pores are visible near its orbital margin. Supraorbital bones are absent.

There are three trapezoidal suborbitals (Figs. 2–4). The anterior two are large and broad, arranged in a dorsoventral row; and the posterior suborbital is small, deep and narrow, positioned between the preopercle and the dermopterotic.

The sclerotic bones are partly discernible near the orbital rim (IVPP V19993), but their number cannot be counted because of poor state of preservation.

### Palate and hyoid arch

The median parasphenoid, often exposed through the orbit, is elongate, bearing a well-developed ascending ramus and a short basipterygoid process on each side of its middle-posterior portion. Teeth are absent on the ventral margin of this bone, as commonly in other early ginglymodians (Olsen & McCune, 1991; Wenz, 1999; Bürgin, 2004; López-Arbarello et al., 2011). A possible right vomer is positioned anterior to the parasphenoid with several teeth discernable on its oral margin. Elements of the palatoquadrate are partly exposed through the orbit as well, but the sutures between them cannot be identified. Two small, elongate bones exposed near the maxilla probably present dermopalatines, medially covered with long, conical teeth (Fig. 4A). The majority of the quadrate (except the condyle) is laterally covered by the fourth infraorbital and the quadratojugal (Fig. 2) and its complete outline is still unknown. The splint-like quadratojugal (Figs. 2– 3) rests along the dorsal margin of the ventral arm of preopercle and contacts the quadrate medially. The hypohyal and the anterior ceratohyal are exposed ventral to the lower jaws in many specimens (Figs. 2– 4). The former is nearly square, and the latter elongate, slightly constricted at its middle portion. The posterior ceratohyal, hyomandibula and symplectic are not exposed.

### Jaws

The upper jaw includes a premaxilla and a maxilla. A supramaxilla is absent. The premaxilla has a horizontally expanded oral region and a deep, posterodorsally directed nasal process (Fig. 4A). Six long, conical teeth are present along the oral margin of the premaxilla. It is still unknown if the olfactory nerve pierces the nasal process of this bone because of the overlay of the rostral and nasals.

The maxilla has a peg-like, medially-directed anterior process and a blade-like posterior portion (Figs. 2– 4). It is relatively short, ending below the anterior orbital margin, with a slightly convex posterior margin and a nearly straight oral margin. The maxilla lacks teeth along its oral margin in available specimens. Considering the well-preserved taphonomic nature of these fossils, we believe that the maxilla is edentulous.

The lower jaw is roughly triangular in lateral view, with a prominent coronoid process. The maximum height is about 40% of its length. The dentary is large and wedge-shaped, with a slightly convex dorsal margin and a concave ventral margin. Large conical teeth are present on the anterior portion of this bone. The sensory pores in the dentary are large, resembling the condition in *Kyphosichthys* (Xu & Wu, 2012; Sun & Ni, 2018). The angular is roughly trapezoidal, being about half of the length of the dentary. The supra-angular is small and elongate, contacting the dentary anteriorly and the angular ventrally. Small coronoid bones are exposed medial to the dentary, but their number cannot be counted. Sharp conical teeth are present on the oral margins of these coronoids (Fig. 4B).

The preopercle is crescent-shaped, having a narrow dorsal portion and a slightly expanded anteroventral portion (Figs. 2– 4). The dorsal tip of the preopercle is separated from contact with the dermopterotic by a suborbital, which bears no sensory canal or pores. The preopercular sensory canal is slightly closer to the anterior margin of the preopercle than to the posterior one (Figs. 2,  4), and runs dorsoventrally through the entire length of the preopercle. Some pores near the posterior margin of the preopercle probably represent the posterior diverticulae of this canal (Fig. 3). The opercle is roughly trapezoidal, 1.75 times deeper than long. The subopercle is sickle-shaped, bearing a relatively short triangular anterodorsal process. Excluding this process, the subopercle is about 30% the depth of opercle. The interopercle is small and triangular. It tapers anteroventrally and nearly reaches the posterior end of the lower jaw.

The median gular, exposed in IVPP V19992 (Figs. 4B) and 19993 ( 4C), is sub-circular and about half of the length of the lower jaw with concentric ridges on the surface of this bone. There are six pairs of branchiostegal rays (Fig. 2B). They are elongate bones, increasing in length and width posteriorly.

### Girdles and fins

A posttemporal, a supracleithrum, a cleithrum and two postcleithra are discernible on each side of the pectoral girdle (Figs. 2B, 2B). The posttemporal is sub-triangular and tapers medially with its anterior portion slightly overlapped by the extrascapular. The left posttemporal is separated from contact with the right one by a median scale. The supracleithrum is deep and anteriorly inclined with its anterior portion overlapped by the opercle. The lateral line enters the supracleithrum from the posttemporal, obliquely penetrates the dorsal portion of this bone, and posteriorly enters the scales. The cleithrum is large and sickle-shaped, with the horizontal branch nearly equal to the vertical branch in length. A series of small denticles is present along the ridge between the branchial and lateral surfaces of this bone. There are two postcleithra. The dorsal is rhombic, nearly as deep as the supracleithrum, and the ventral trapezoidal, half the depth of the dorsal.

The pectoral fins insert low on the body, and each bears nine distally segmented rays, preceded by two or three basal fulcra and a series of fringing fulcra. The pelvic girdles are not exposed. The pelvic fins insert at the 8th or 9th vertical scale row. Each bears five distally segmented rays, preceded by three basal fulcra and a series of fringing fulcra. In both paired fins, the first ray is unbranched, and the remaining rays branched distally.

The dorsal fin originates above the 20th vertical scale row. It is composed of 9–11 principal rays. The first ray is unbranched, preceded by three basal fulcra and a series of fringing fulcra; and the remaining branched distally. The anal fin originates below the 14th or 15th vertical scale row and has eight principal rays. The first is unbranched, preceded by a short rudimentary ray, one or two basal fulcra and a series of fringing fulcra; the remaining rays branched distally. Occasionally, the rudimentary ray is absent in some specimens.

**Figure 5:**
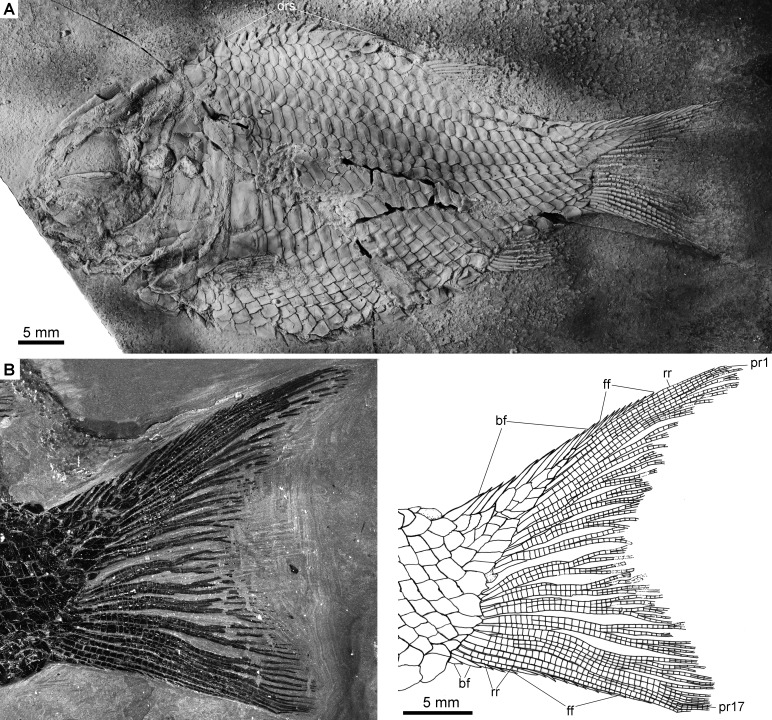
Complete skeleton of IVPP V22922 and caudal fin of IVPP V19992. *Fuyuanichthys wangi***gen*.* et sp. nov.** (A) Complete skeleton, IVPP V22922, coated with ammonium chloride. (B) Caudal fin, IVPP V19992, immersed in water when photographed.

The caudal fin is hemi-heterocercal with a moderately concaved profile (Fig. 5B). It bears 16–18 principal rays, of which eight or nine are present in the dorsal lobe. The marginal principal rays are unbranched and the middle rays are branched up to four times. The articulations between the segments of rays are straight. In addition, there are eight to ten basal fulcra and one rudimentary ray in the dorsal lobe, and two basal fulcra and two rudimentary rays in the ventral lobe. The basal fulcra are elongate, leaf-like elements. Small, elongate fringing fulcra are present at the leading margins of both lobes.

All rays and fulcra are covered with a layer of ganoine, and their surfaces are largely smooth, lacking any tubercles.

### Scales

The body is fully covered with rhomboid scales. The scales are arranged in 29–31 vertical rows along the lateral line. In addition, there are 8–9 vertical rows of scales extending into the epaxial lobe of the caudal fin. The ridge scales anterior to the dorsal fin are conspicuous with well-developed spines (Fig. 5A). The scales in the anterior flank region are 1.2 times deeper than wide, and they gradually become shorter and smaller dorsally, ventrally and posteriorly. The scales are smooth with a straight posterior margin. Pegs and anterodorsal extensions are exposed on some scales in the anterior flank region (Appendix S1: Fig. 1).

## Phylogenetic Results

**Figure 6:**
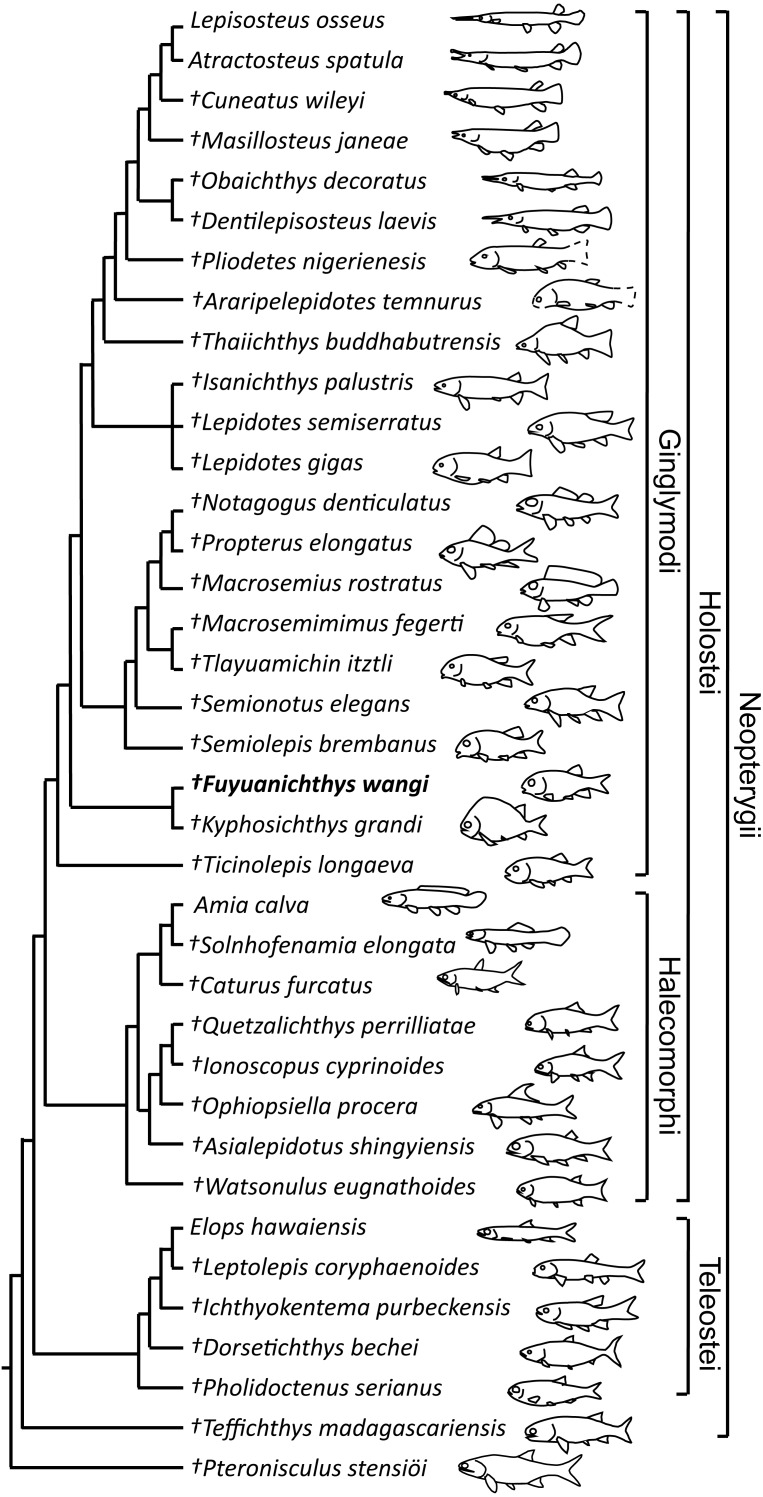
Strict consensus of three most parsimonious trees. Strict consensus of three most parsimonious trees (tree length = 478 steps, consistency index = 0.5021, retention index = 0.7503), illustrating the phylogenetic relationships of *Fuyuanichthys wangi* gen*.* et sp. nov. within the Neopterygii. For character descriptions and codings for the sampled taxa, see Supplemental Information 1.

The phylogenetic analysis resulted in three most parsimonious trees (tree length = 478 steps, consistency index = 0.5021, retention index = 0.7503), the strict consensus of which is illustrated in Fig. 6. *Fuyuanichthys* is recovered as a sister taxon to *Kyphosichthys* at the ginglymodian stem; the *Fuyuanichthys*-*Kyphosichthys* clade possesses the following synapomorphies of Ginglymodi: (1), presence of two anterior infraorbitals; (2), presence of six infraorbitals (including four infraorbitals between the antorbital and the infraorbital at the posteroventral corner of the orbit); (3), presence of no more than nine pairs of branchiostegal rays (independently derived in *Teffichthys*); and (4), presence of a relatively short lower jaw (mandibular length no more than 43% of head length).

*Fuyuanichthys* shares four derived features with *Kyphosichthys*: (1), the paired extrascapulars separated from each other by posterior extensions of parietals (independently derived in *Macrosemimimus* and macrosemiids); (2), the quadrate laterally covered by infraorbitals (independently derived in *Thaiichthys*, *Araripelepidotes* and lepisosteids); (3), presence of suborbital bones between the preopercle and the dermopterotic (independently derived in *Tlayuamichin*, *Watsonulus* and some teleosts); and (4), presence of a complete row of elongated scales between the last lateral line scale and the uppermost caudal fin ray (independently derived in *Pholidoctenus*).

The *Fuyuanichthys*-*Kyphosichthys* clade is more derived than *Ticinolepis* (the most basal ginglymodian) in sharing six derived features with the Semionotiformes-Lepisosteiformes clade (=Neoginglymodi of López-Arbarello & Sferco, 2018): (1), presence of a relatively long parietal (independently derived in some other neopterygians); (2), a subrectangular dermopterotic not substantially tapered anteriorly or widened posteriorly; (3), a dermopterotic no longer than the parietal; (4), a lacrimal deeper than long; (5), a relatively narrow posttemporal not reaching the midline; and (6) absence of a presupracleithrum. However, this clade lacks some derived features of Semionotiformes shared with Lepisosteiformes, e.g., presence of eight or more infraorbitals (including six or more infraorbital bones between the antorbital and the infraorbital at the posteroventral corner of the orbit), and absence of a supramaxillary processes on the maxilla.

## Discussions

*Fuyuanichthys* represents the first convincing record of Ginglymodi in the late Middle Triassic (Ladinian) of China. The previously alleged ginglymodian *Asialepidotus* from the same fossil beds has recently been revised as an ionoscopiform halecomorph (Xu & Ma, 2018). Jin (2006), in a review of Triassic fishes from China, identified three ginglymodians (*Eosemionotus* sp., *Archaeosemionotus* sp. and an unnamed marosemiid) from the Ladinian marine deposits of Yunnan and Guizhou, and argued that the Ladinian fish assemblage from China is closely related to the contemporaneous fish assemblages from the Monte San Giorgio area of Switzerland and Lombardy of Italy in the western Palaeotethys. However, these ginglymodian taxa have never been described or illustrated. We have not found *Eosemionotus*, *Archaeosemionotus* or marosemiids in Southwest China during our multiple-year field collection. The new fossil specimens described herein cannot be referred to the ginglymodian taxa mentioned by Jin (2006). *Fuyuanichthys* differs from *Eosemionotus* (Schultze & Möller, 1986; Bürgin, 2004) in having a median gular, a pair of frontals (vs. fused into a median plate in *Eosemionotus*), a quadrate laterally covered by infraorbitals (vs. exposed in *Eosemionotus*), three suborbitals (vs. absent in *Eosemionotus*), a relatively short dermopterotic (vs. long in *Eosemionotus*), the separation of extrascapulars by the parietals (vs. extrascapulars contacting medially in *Eosemionotus*), and a caudal fin composed of 16–18 principal rays (vs. 13–15 in *Eosemionotus*). As for *Archaeosemionotus*, this taxon has no longer been considered as a semionotid ginglymodian but a basal halecomorph (López-Arbarello & Sferco, 2018). Additionally, results of our analysis suggest that *Fuyuanichthys* is phylogenetically distant from Marosemiidae (Bartram, 1977), although it independently evolved a few traits similar to those in the latter family, such as presence of separated extrascapulars and absence of a supramaxilla.

The discovery of *Fuyuanichthys* fills the previous deficiency of ginglymodian fishes in the Middle Triassic of Guizhou and extends the geological range of this clade from the early Middle Triassic (Anisian, ∼244 Ma) of Luoping (*Sangiorgioichthys sui*, *Kyphosichthys grandei*) and Panxian (*S. yangjuanensis*) into the late Middle Triassic (Ladinian, ∼240 Ma) of Fuyuan and Xingyi in South China. *Fuyuanichthys* possesses a unique combination of features that easily distinguishes it from other ginglymodians: (1), absence of supraorbitals (independently lost in some macrosemiids); (2), absence of a supramaxilla (independently lost in macrosemiids and crownward lepisosteiforms); (3) absence of teeth in the maxilla (independently lost in *Tlayuamichin* and several other ginglymodians); (4), presence of two vertical rows of suborbitals; and (5), presence of a median gular (also present in *Kyphosichthys*, but absent in other ginglymodians; the loss of the median gular in the basal ginglymodian *Ticinolepis* and crownward ginglymodians appears independently derived). This unique combination of features makes *Fuyuanichthys* inappropriate to be classified in any ginglymodian families previously known, and it likely represents a new family of this clade. As such, the new finding provides an important addition to our understanding of the morphological diversification of early ginglymodians.

*Fuyuanichthys* represents one of the smallest known holosteans in the Xingyi biota, with a maximum standard length (SL) of ∼75 mm. With regard to body size, it is similar to *Sinoeugnathus* (Su, 1959) and *Subortichthys* (Ma & Xu, 2017; SL = 72 mm) among the Middle Triassic holosteans, and significantly smaller than the contemporary halecomorph *Asialepidotus* (Xu & Ma, 2018; SL = 273 mm). In Luoping biota, halecomorphs have a maximum SL up to 360 mm (*Robustichthys*, Xu, Gao & Finarelli, 2014), and ginglymodians (*Kyphosichthys*, Xu & Wu, 2012; *Sangiorgioichthys sui*, López-Arbarello et al., 2011) are small-sized fishes with a SL of no more than 125 mm. Additionally, in the Panxian biota, the ginglydomian (*S. yangjuanensis*, Chen et al., 2014; SL = 56 mm) is quite smaller than the halecomorph *Panxianichthys* (Xu & Shen, 2015; Sun et al., 2017; SL = ∼175 mm). It appears that the Ginglymodi have a narrower range of body sizes than the Halecomorphi from the Middle Triassic of South China in the eastern Palaeotethys. The largest holosteans in this age are represented by halecomorphs from the Middle Triassic of South China. In comparison, a somewhat different phenomenon is present in the Middle Triassic holostean assemblages from the Monte San Giorgio area of Switzerland and Lombardy of Italy in the western Palaeotethys, in which, ginglymodians have a range of SL from 50 mm (*Eosemionotus*, Schultze & Möller, 1986; Bürgin, 2004) to 250 mm (*Ticinolepis*, López-Arbarello et al., 2016), and halecomorphs are generally small-sized fishes with a SL of no more than 110 mm (*Eoeugnathus*, Herzog, 2003; *Archaeosemionotus*, López-Arbarello, Stockar & Bürgin, 2014; and *Allolepidotus*, Lombardo, 2001; Sun et al., 2017). No large-sized halecomorphs have been known from the Middle Triassic of the western Palaeotethys. Further studies are needed to clarify if this is caused by sampling or taphonomic bias.

As listed above, *Fuyuanichthys* differs from other ginglymodians in many features, but it shares with *Kyphosichthys* and *Sangiorgioichthys sui* a noteworthy feature, a pair of extrascapulars separated from each other by the posterior portions of parietals with the supratemporal commissural canal running transversely through both extrascapulars and parietals. A similar condition is present in marosemiids (Bartram, 1977). Some paleoichthyologists (López-Arbarello et al., 2011; Sun & Ni, 2018) suggested that *Sangiorgioichthys* and *Kyphosichthys* primitively have two pairs of extrascapulars, and the medial extrascapular had fused with the parietal. The same hypothesis appears applicable for marosemiids and *Fuyuanichthys*. According to recent analyses (Sun & Ni, 2018; López-Arbarello & Sferco, 2018; and us herein), *Sangiorgioichthys*, *Kyphosichthys* and *Fuyuanichthys* are phylogenetically distant from Marosemiidae, and the fusion of the medial extrascapular with the parietal in *Sangiorgioichthys*, *Kyphosichthys* and *Fuyuanichthys* most likely represents convergent evolution with that in the Marosemiidae. However, it is still unknown whether this fusion in the three basal ginglymodians from Southwest China has evolved independently or not. If *Sangiorgioichthys*, *Kyphosichthys* or *Fuyuanichthys* are closely related, as suggested respectively by Sun & Ni (2018) and us herein, the fusion of the medial extrascapular with the parietal appears to have happened in the common ancestor of these three taxa.

Sun & Ni (2018) grouped *Kyphosichthys*, *Sangiorgioichthys sui*, *S. aldae* and *Luoxiongichthys* into their coined family Kyphosichthyidae, and listed three synapomorphies for this family: (1) a triangular suborbital laterally covering the quadrate; (2) infraorbitals at the ventral orbital rim subtriangular, broader ventrally, and about twice deeper than long; and (3) absence of a foramen for the olfactory nerve in the nasal process of the premaxilla. However, the first feature is actually absent in *Kyphosichthys*; instead, the quadrate is laterally covered by a broad infraorbital in this taxon, as in *Fuyuanichthys*. Although the quadrate of *Luoxiongichthys* is also laterally covered by a suborbital, this suborbital is trapezoid (Wen et al., 2012). Indeed, a triangular suborbital laterally covering the quadrate is only present in *Sangiorgioichthys*, as a diagnostic feature of this genus (López-Arbarello & Alvarado-Ortega, 2011). Additionally, it appears that the second feature was not appropriately coded. The ventral infraorbitals are not subtriangular but quadrangular in this alleged family; they are notably deep (twice deeper than long) in *Kyphosichthys* but are relatively dorsoventrally short (1.3–1.7 deeper than long) in other three taxa. As for the last feature, a foramen for the olfactory nerve is absent in the nasal process of the premaxilla of *Luoxiongichthys* (G-H Xu’s, pers. obs., 2018) but is actually present in *Sangiorgioichthys sui* (G-H Xu & X-Y Ma’s, pers. obs., 2018), as in most ginglymodians (except *Ticinolepis* and marosemiids). The condition is still unknown in *S. aldae* (Tintori & Lombardo, 2007) and *Kyphosichthys* (Xu & Wu, 2012; Sun & Ni, 2018) because of poor state of preservation. Therefore, the three features above should not be considered as kyphosichthyid synapomorphies. A revision of Sun & Ni’s (2018) Kyphosichthyidae is badly needed (López-Arbarello & Sferco, 2018). Based on our preliminary studies, *Luoxiongichthys* is phylogenetically distant from *Kyphosichthys*, although they have a similarly deep body. A redescription and revision of *Luoxiongichthys* will be presented in the future.

## Conclusion

The discovery of *Fuyuanichthys wangi* from the renowned Ladinian Xingyi biota fills the previous deficiency of ginglymodians at this age in China and provides an important addition to our understanding of the morphological diversification of early ginglymodians. *Fuyuanichthys* is unusual among the Middle Triassic ginglymodians in having a median gular, short and edentulous maxillae and two vertical rows of suborbitals, and lacking any supramaxilla and supraorbitals. As one of the smallest known ginglymodians, the new finding further supports that the Middle Triassic Ginglymodi have a relatively small range of body sizes compared with the Halecomorphi from the same ecosystems in China. Results of a phylogenetic analysis recovers *Fuyuanichthys* as a sister taxon to *Kyphosichthys* at the ginglymodian stem, and provides new insights into the early evolution of this clade.

##  Supplemental Information

10.7717/peerj.6054/supp-1 Supplemental Information 1 Characters, data matrix and Supplementary figures

## Anatomical abbreviations:

ang: angular
ao: antorbital
ap: ascending process
bf: basal fulcrum
bp: basipterygoid process
br: branchiostegal ray
cha: anterior ceratohyal
cl: cleithrum
co: coronoid
den: dentary
dp: dermopalatine
dpt: dermopterotic
drs: dorsal ridge scale
dsp: dermosphenotic
es: extrascapular
ff: fringing fulcrum
fr: frontal
gu: gular
hh: hypohyal
io: infraorbital
iop: interopercle
le: lateral ethmoid
mx: maxilla
na: nasal
op: opercle
pa: parietal
pas: parasphenoid
pcl: postcleithrum
pr: principle fin ray
pmx: premaxilla
pop: preopercle
pt: posttemporal
qj: quadratojugal
qu: quadrate
ro: rostral
rr: rudimentary fin ray
sa: supra-angular
scl: supracleithrum
so: suborbital
sop: subopercle
vo: vomer

## Additional Information and Declarations

### Data Availability

The following information was supplied regarding data availability: The raw data is available as Supplemental Information 1.

### New Species Registration

The following information was supplied regarding the registration of a newly described species: Publication LSID: urn:lsid:zoobank.org:pub:69D69D4F-1F36-472D-BBA5-BA37E22E360B Genus name, Fuyuanichthys: urn:lsid:zoobank.org:act:41FDD725-A826-4214-BFDC-3F455B4C48A9. Species name, *Fuyuanichthys wangi*: urn:lsid:zoobank.org:act:99A08E2C-EC59-42F0-8EF8-62778D20D00F.
