# Supplementary material for: Fuyuanichthys wangi gen. et sp. nov. from the Middle Triassic (Ladinian) of China highlights the early diversification of ginglymodian fishes
Source: PeerJ. 2018 Dec 20;6:e6054. doi: 10.7717/peerj.6054 (PMC6304272; doi:10.7717/peerj.6054)
Supplement: Supplemental Information 1 [file peerj-06-6054-s001.doc]

SUPPLEMENTARY DATA

***Fuyuanichthys wangi* gen. et sp. nov. from the Middle Triassic (Ladinian) of China highlights the early diversification of ginglymodian fishes**

Guang-hui Xu^1,2^, Xin-ying Ma^1,2,3^ and Yi Ren^1,2,3^

^1^Key Laboratory of Vertebrate Evolution and Human Origins of Chinese Academy of Sciences, Institute of Vertebrate Paleontology and Paleoanthropology, Chinese Academy of Sciences, Beijing 100044, China

^2^CAS Center for Excellence in Life and Paleoenvironment, Beijing, 100044, China

^3^Univesity of Chinese Academy of Sciences, Beijing 100049, China

Part A. Material examined and references.

Character codes for *Fuyuanichthys* gen. nov. were based on our own examinations of specimens, and those for other taxa were based on the following literature (with our examinations of some specimens):

*Amia calva* and *Solnhofenamia elongate* (Grande and Bemis, 1998);

*Araripelepidotes temnurus* (Maisey, 1991; Thies, 1996);

*Asialepidotus xingyiensis* (Xu and Ma, 2018);

*Atractosteus spatula*, *Cuneatus wileyi*, *Dentilepisosteus laevis*, *Lepisosteus osseus*, *Masillosteus janeae*, and *Obaichthys decoratus* (Grande, 2010);

*Caturus furcatus* (Patterson, 1975; Lambers, 1992; Grande and Bemis, 1998; FMNH UC2057);

*Dorsetichthys* (‘*Pholidophorus*’) *bechei* (Patterson, 1975; Grande and Bemis, 1998; Arratia, 2013);

*Elops hawaiensis* (Forey, 1973);

*Ichthyokentema purbeckensis* (Griffith and Patterson, 1963);

*Ionoscopus cyprinoides* (Grande and Bemis, 1998; Maisey,1999; FMNH P15472);

*Isanichthys palustris* (Cavin and Suteethorn, 2006);

*Kyphosichthys* grandei (Xu and Wu, 2012; Sun and Ni, 2018);

*Lepidotes gigas* (Thies, 1989; López-Arbarello, 2012);

*L. semiserratus* (Stensiö, 1932; Rayner, 1948; Patterson, 1975);

*Leptolepis coryphaenoides* (Patterson 1975);

*Macrosemius rostratus*, *Notagogus denticulatus* and *Propterus elongatus* (Bartram, 1977);

*Macrosemimimus fegerti* (Schröder et al., 2012);

*Ophiopsiella* (‘*Ophiopsis*’) *procera* (Bartram, 1975; Lane and Ebert, 2015; FMNH UC2037);

*Pholidoctenus serianus* (Arratia, 2013);

*Pliodetes nigeriensis* (Wenz, 1999);

*Pteronisculus stensioi* (Nielsen, 1942);

*Quetzalichthys perrilliatae* (Alvarado-Ortega and Espinosa-Arrubarrena, 2008);

*Semiolepis brembanus* (Lombardo and Tintori, 2008); *Semionotus elegans* (Olsen and McCune 1991);

*Teffichthys* (‘*Perleidus*’) *madagascariensis* (Lehman, 1952; Marramà et al., 2017; NHMUK P16247, 16248, 19580–19584, 19587–19592, 19595–19599, 19603–19620, 19622, 19623);

*Thaiichthys buddhabutrensis* (Cavin et al., 2013);

*Ticinolepis longaeva* (López-Arbarello et al., 2016);

*Tlayuamichin itztli* (López-Arbarello and Alvarado-ortega, 2011);

*Watsonulus eugnathoides* (Olsen, 1984; Grande and Bemis, 1998).

Institutional abbreviations: NHMUK, Natural History Museum, London, UK; FMNH, Field Museum of Natural History, Chicago, USA.

Part B. Characters and character states

**Neurocranium**

1. Post-temporal fossa

(Grande, 2010, Xu and Gao, 2011; Xu and Wu, 2012; Xu et al., 2012, 2014, 2015; López-Arbarello, 2012; Cavin et al., 2013; Deesri et al., 2014, 2016; Giles et al., 2017)

- - 1. Absent
    2. Present

1. Vagal foramen

(Modified from Gardiner et al. 1996; Cavin and Suteethorn 2006; Hurley et al. 2007; López-Arbarello, 2012; Giles et al., 2017; López-Arbarello and Sferco, 2018. This character can only be coded when separate braincase ossifications can be identified.)

- - 1. At the exoccipital margin
    2. Enclosed by exoccipital
    3. Unrelated to exoccipital

1. Intercalar

(Olsen, 1994; Gardiner et al., 1996; Grande, 2010; López-Arbarello, 2012; Cavin et al., 2013; Cavin et al., 2013; Xu et al., 2014; Xu and Shen, 2015; Xu and Zhao, 2016; Deesri et al., 2014, 2016; Giles et al., 2017; López-Arbarello and Sferco, 2018. This character can only be coded when separate braincase ossifications can be identified.)

- - 1. Present
    2. Absent

1. Membranous outgrowth of intercalar

(Gardiner et al. 1996; Hurley et al. 2007; Giles et al., 2017; López-Arbarello and Sferco, 2018. This character can only be coded when separate braincase ossifications can be identified.)

- - 1. Absent
    2. Present

1. Basisphenoid

(Cavin, 2010; López-Arbarello, 2012; Cavin et al., 2013; Deesri et al., 2014, 2016; Giles et al., 2017; López-Arbarello and Sferco, 2018)

- - 1. Present
    2. Absent or very reduced

1. Opisthotic

(Grande and Bemis, 1998; Cavin and Suteethorn 2006; Hurley et al. 2007; Grande 2010; López-Arbarello, 2012; Cavin et al., 2013; Deesri et al., 2014, 2016; Xu et al., 2014; Giles et al., 2017; Ma and Xu, 2017; Xu and Ma, 2018; López-Arbarello and Sferco, 2018. This character can only be coded when separate braincase ossifications can be identified.)

- - 1. Present
    2. Absent

1. Pterotic

(Grande and Bemis, 1998; Xu and Wu, 2012; Brito and Alvardo-Ortega, 2013; Xu et al., 2014; Xu and Shen, 2015; Sun et al., 2017; Giles et al., 2017; Ma and Xu, 2017; Xu and Ma, 2018. This character can only be coded when separate braincase ossifications can be identified.)

- - 1. Present
    2. Absent

1. Supraotic

(Gardiner et al., 1996; López-Arbarello and Sferco, 2018. This character can only be coded when separate braincase ossifications can be identified.)

1. Absent
2. Present
3. Ethmoid ossification

(Grande, 2010; Xu et al., 2014; Xu and Shen, 2015; Sun et al., 2017. This character can only be coded when separate braincase ossifications can be identified.)

1. Present
2. Absent
3. Anterior extent of parasphenoid tooth patch

(Grande and Bemis, 1998; Xu et al., 2014; Xu and Shen, 2015; Ma and Xu, 2017; Xu and Ma, 2018; López-Arbarello and Sferco, 2018)

- - 1. Extends well anterior to the lateral ascending arms of parasphenoid
    2. Short, does not extend anterior to the lateral ascending arms
    3. Parasphenoid tooth patch absent

1. Parasphenoid

(Coates, 1999; Gardiner et al., 2005; Friedman, 2007, Xu and Gao, 2011; Xu et al., 2012, 2014; Xu and Zhao, 2016; Giles et al., 2017; López-Arbarello and Sferco, 2018)

- - 1. Terminates at/anterior to ventral otic fissure
    2. Extends across ventral otic fissure
    3. Extends to basioccipital

1. Intercalar/parasphenoid contact

(Gardiner et al., 1996; Xu et al., 2014; Xu and Shen, 2015; Sun et al., 2017; Xu and Ma, 2018. This character can only be coded when separate braincase ossifications can be identified.)

- - 1. Absent
    2. Present

1. Internal carotid foramen on parasphenoid

(Gardiner et al., 1996; Hurley et al. 2007; Xu and Wu, 2012; Xu et al., 2012, 2014; Xu and Shen, 2015; Xu et al., 2015; Xu and Zhao, 2016; Giles et al., 2017; Ma and Xu, 2017; Xu and Ma, 2018)

- - 1. Absent
    2. Present

1. Efferent pseudobranchial foramen on parasphenoid

(Gardiner et al., 1996; Hurley et al. 2007; Xu and Wu, 2012; Xu et al., 2012, 2014; Xu and Shen, 2015; Xu et al., 2015; Xu and Zhao, 2016; Sun et al., 2017; Giles et al., 2017; Ma and Xu, 2017; Xu and Ma, 2018)

- - 1. Absent
    2. Present

1. Parasphenoid ascending process

(Gardiner et al., 1996)

1. Does not contact sphenotic
2. Contacts sphenotic
3. Sphenotic with small dermal component

(Grande, 2010; Xu and Wu, 2012; Xu et al., 2012, 2014, 2015; Xu and Shen, 2015; Sun et al., 2017; Giles et al., 2017; Ma and Xu, 2017; Xu and Ma, 2018; López-Arbarello and Sferco, 2018)

- - 1. Absent
    2. Present

1. Posterior myodome

(Gardiner and Schaeffer, 1989; Gardiner et al. 1996; Hurley et al. 2007;Coates, 1999; Xu and Gao, 2011; Xu and Wu, 2012; Xu et al. 2012, 2014, 2015; López-Arbarello, 2012; Xu and Zhao, 2016; Giles et al., 2017; López-Arbarello and Sferco, 2018)

- - 1. Present
    2. Absent

1. Lateral process of basioccipital

(Grande, 2010, Cavin et al., 2013; Deesri et al., 2014, 2016)

- - 1. Absent
    2. Present

1. Posterior extent of exoccipitals in adult-sized individuals

(Grande and Bemis, 1998; Xu et al., 2014; Xu and Shen, 2015; Sun et al., 2017; Ma and Xu, 2017; Xu and Ma, 2018; Ebert, 2018)

- - 1. Reaches posterior margin of occiput
    2. Does not reach posterior margin of occiput

1. Supraoccipital bone

(Grande, 2010; López-Arbarello, 2012; Arratia, 2013; Cavin et al., 2013; Deesri et al., 2014, 2016; Xu et al., 2014; Xu and Shen, 2015; Sun et al., 2017; Giles et al., 2017; Xu and Ma, 2018; López-Arbarello and Sferco, 2018. This character can only be coded when separate braincase ossifications can be identified.)

- - 1. Absent
    2. Present

**Snout and skull roof**

1. Elongation of the rostral region anterior to the lower jaw symphysis

(Grande, 2010; López-Arbarello, 2012; Cavin et al., 2013; Deesri et al., 2014, 2016; López-Arbarello and Sferco, 2018)

1. Extends anterior to the dentary symphysis by less than 20% of mandibular length
2. Extends well anterior to the dentary symphysis by more than 50% of mandibular length
3. Rostral bone

(Gardiner et al., 1996; Grande and Bemis, 1998; Xu and Wu, 2012; Xu et al., 2012, 2014; Brito and Alvardo-Ortega, 2013; Xu and Shen, 2015; Sun et al., 2017; Giles et al., 2017; Ma and Xu, 2017; Xu and Ma, 2018)

- - - 1. A deep cap on tip of snout
      2. Of moderate size, sub-circular
      3. Much reduced, sub-triangular, elongate, V-shaped or tube-like
      4. Lost as an autogenous bone

1. Dermal bones of the skull ornamented with firmly anchored, pointed conical denticles

(Grande, 2010; López-Arbarello, 2012; Cavin et al., 2013; Deesri et al., 2016; López-Arbarello and Sferco, 2018)

- - - 1. Absent
      2. Present

1. Parietal length

(Grande and Bemis, 1998; Xu et al., 2014; Xu and Shen, 2015; Sun et al., 2017; Ma and Xu, 2017; Xu and Ma, 2018; Ebert, 2018; López-Arbarello and Sferco, 2018)

1. Short, with a width-to-length ratio range well exceeding 0.90
2. Relatively long, with a width-to-length ratio range not exceeding 0.90
3. Ratio of frontal/parietal length

(Modified from López-Arbarello, 2012; Cavin et al., 2013; Deesri et al., 2016; López-Arbarello and Wencker, 2016; Sun et al., 2017; López-Arbarello and Sferco, 2018)

1. 1.5 or more
2. Less than 1.5
3. Frontal width in adult-sized individuals

(Modified from Grande and Bemis, 1998; López-Arbarello, 2012; Xu et al., 2014; Xu and Shen, 2015; López-Arbarello and Wencker, 2016; Sun et al., 2017; Ma and Xu, 2017; Xu and Ma, 2018; López-Arbarello and Sferco, 2018)

1. Relatively wide, with a width-to-length ratio of 0.22 to 0.65
2. Relatively narrow, with a width-to-length ratio of no more than 0.21
3. Anterior portion of frontal extends well beyond supraorbitals (pre-supraorbital length of the frontal nearly equaling one-fifth or more the total length of this bone)
4. Absent
5. Present
6. Shape of dermopterotic (=supratemporal+intertemporal)

(Grande and Bemis, 1998; Xu et al., 2014; Xu and Shen, 2015; Sun et al., 2017; Ma and Xu, 2017; Xu and Ma, 2018)

1. Greatly widened posteriorly and tapered anteriorly
2. Subrectangular, not substantially tapered anteriorly or widened posteriorly
3. Dermopterotic length to parietal length

(Gardiner et al., 1996; Grande and Bemis, 1998; Xu et al., 2014; Xu and Shen, 2015; López-Arbarello and Wencker, 2016; Sun et al., 2017; Ma and Xu, 2017; Xu and Ma, 2018)

1. Dermopterotic significantly longer
2. Lengths about equivalent
3. Number of extrascapular bones

(Grande and Bemis, 1998; Xu et al., 2014; Xu and Shen, 2015; Sun et al., 2017; Ma and Xu, 2017; Xu and Ma, 2018; López-Arbarello and Sferco, 2018)

1. One pair
2. Two pairs
3. More than two pairs
4. Extrascapulars separated by posterior extensions of parietals

(López-Arbarello, 2012; López-Arbarello and Wencker, 2016; Sun et al., 2017; López-Arbarello and Sferco, 2018)

1. Absent
2. Present
3. Long and narrow anterorbital portion of frontal

(Modified from López-Arbarello, 2012; López-ArbarelloandWencker, 2016; Sun et al., 2017; López-Arbarello and Sferco, 2018)

1. Absent
2. Present
3. Rostral/frontal contact

(Grande and Bemis, 1998; Xu et al., 2014; Xu and Shen, 2015; Sun et al., 2017; Ma and Xu, 2017; Xu and Ma, 2018)

1. Present
2. Absent
3. Contact relationships of nasals

(Arratia, 2013; Ma and Xu, 2017; Giles et al., 2017; Xu and Ma, 2018; López-Arbarello and Sferco, 2018)

1. Separated by rostral
2. Contacting medially or close to contact medially (not separated by other bones)
3. Separated by frontals
4. Separated by mesoethmoid
5. Nasal contributing to orbital margin

(Xu and Wu 2012; Xu et al. 2014; Xu and Zhao, 2016; Giles et al., 2017; López-Arbarello and Sferco, 2018)

1. Present
2. Absent
3. Frontal contributing to orbital margin
4. Absent
5. Present
6. Commissure between right and left supraorbital canal within frontal

(Grande, 2010; Cavin et al., 2013; Deesri et al., 2016)

1. Absent
2. Present
3. Parietal portion of the supraorbital sensory canal

(Cavin, 2010; López-Arbarello, 2012; Cavin et al., 2013; Deesri et al., 2016; Ma and Xu, 2017; Sun et al., 2017; Xu and Ma, 2018)

- - 1. Present
    2. Absent

1. Junction of supraorbital canal with infraorbital canal exclusively in the dermopterotic (Grande, 2010; Ma and Xu, 2017; Xu and Ma, 2018)
2. Absent
3. Present

**Circumorbital bones**

1. Tube-like canal bearing anterior arm on the antorbital bone.

(Grande, 2010; Xu and Wu, 2012; Brito and Alvardo-Ortega, 2013; Xu et al., 2014; Xu and Shen, 2015; Ma and Xu, 2017; Sun et al., 2017; Giles et al., 2017; Xu and Ma, 2018; López-Arbarello and Sferco, 2018)

1. Absent
2. Present
3. Supraorbital

(Modified from Grande and Bemis, 1998; Brito and Alvardo-Ortega, 2013; Xu et al., 2014; Xu and Shen, 2015; Sun et al., 2017; Giles et al., 2017; Ma and Xu, 2017; Xu and Ma, 2018; López-Arbarello and Sferco, 2018)

- 1. Absent
  2. Present

1. Number of supraorbital bones

(Modified from Grande and Bemis, 1998; Brito and Alvardo-Ortega, 2013; Xu et al., 2014; Xu and Shen, 2015; Sun et al., 2017; Giles et al., 2017; Ma and Xu, 2017; Xu and Ma, 2018; Ebert, 2018; López-Arbarello and Sferco, 2018. *Quetzalichthys* has at least two supraorbitals (Alvarado-Ortega and Espinosa-Arrubarrena, 2008), but the exact number of supraorbitals are still unknown because of incomplete preservation.)

1. Three or more
2. Two
3. Single
4. Circumorbital ring

(Grande, 2010; Cavin et al., 2013; Deesri et al., 2016; Giles et al., 2017; López-Arbarello and Sferco, 2018)

- - 1. Supraorbitals do not contact infraorbital series at the anterior rim of the orbit
    2. Supraorbitals contact infraorbitals, closing the orbit

1. Supraorbital/antorbital contact
   - - 1. Absent
       2. Present
2. Antorbital/frontal contact
   - - 1. Absent
       2. Present
3. Infraorbital/dermopterotic contact
   - - 1. Absent
       2. Present
4. Anterior infraorbitals

(Cavin and Suteethorn 2006; Grande, 2010; Xu and Wu, 2012; López-Arbarello, 2012; Xu et al. 2014, 2015; Xu and Zhao, 2016; Giles et al., 2017; Ma and Xu, 2017; Xu and Ma, 2018; López-Arbarello and Sferco, 2018. The ‘anterior infraorbitals’ refer to the infraorbital bones that locate posterior to the antorbital and do not contribute to the orbital margin (Wenz, 1999; López-Arbarello, 2012).

1. Absent
2. Present
3. Number of anterior infraorbitals

(Cavin and Suteethorn 2006; Grande, 2010; Xu and Wu, 2012; López-Arbarello, 2012; Xu et al. 2014, 2015; Xu and Zhao, 2016; Giles et al., 2017; Ma and Xu, 2017; Xu and Ma, 2018; López-Arbarello and Sferco, 2018)

- - 1. One or two
    2. Three
    3. Four or more

1. Contact relationships of the anterior-most supraorbital with anterior infraorbitals
2. Supraorbital does not contact anterior infraorbitals
3. Supraorbital contacts a single anterior infraorbital
4. Supraorbital contacts two or more anterior infraorbitals
5. Contact relationships of the frontal with anterior infraorbitals
6. frontal does not contact anterior infraorbitals
7. frontal contacts anterior infraorbitals
8. Contact relationships of infraorbital bone at posteroventral corner of orbit with preopercle

(Modified from López-Arbarello, 2012; Xu and Ma, 2018; López-Arbarello and Sferco, 2018)

- - 1. Absent
    2. Present

1. Posterior notch of second infraorbital for supramaxilla

(Xu et al., 2014; Xu and Shen, 2015; Sun et al., 2017)

1. Absent
2. Present
3. Antorbital contributing to orbital margin

(Xu et al., 2012; Xu et al., 2014; Xu and Shen, 2015; Xu and Zhao, 2016; Sun et al., 2017; López-Arbarello and Sferco, 2018)

1. Present
2. Absent
3. Lower margin of last infraorbital inclined posterodorsally

(Gardiner et al., 1996; Alvarado-Ortega and Espinosa-Arrubarrena, 2008; Xu et al., 2014; Xu and Shen, 2015; Ma and Xu, 2017; Xu and Ma, 2018)

1. Absent
2. Present
3. Size of last infraorbital

(Modified from Grande and Bemis, 1998)

1. Small, relatively narrow
2. Large, posteriorly expanded
3. Number of infraorbitals between antorbital and dermosphenotic

(Gardiner and Schaeffer, 1989; Xu et al., 2012; Xu et al., 2014; Xu and Shen, 2015; Ma and Xu, 2017; Xu and Ma, 2018)

1. Two or three
2. Four or five
3. Six or seven
4. Eight or more
5. Number of the infraorbital bones between the antorbital and the infraorbital at posteroventral corner of the orbit

(Modified from Gardiner et al., 1996)

- - 1. Single
    2. Two or three
    3. Four or five
    4. Six or more

1. Lachrymal

(Modified from Grande and Bemis, 1998; Xu et al., 2014; Xu and Shen, 2015; Sun et al., 2017; Ma and Xu, 2017; Xu and Ma, 2018)

- - 1. Longer than deep
    2. Deeper than long

1. Lachrymal relative to orbit in size

(Modified from Grande and Bemis, 1998; Xu et al., 2014; Xu and Shen, 2015; Sun et al., 2017; Ma and Xu, 2017; Xu and Ma, 2018)

1. Significantly smaller than orbit
2. Nearly equal to orbit in size
3. Inner orbital flange of dermosphenotic

(Grande and Bemis, 1998; Brito and Alvardo-Ortega, 2013; Xu et al., 2014; Xu and Shen, 2015; Sun et al., 2017; Ma and Xu, 2017; Xu and Ma, 2018)

1. Smooth, without sensory canal
2. Bearing sensory canal tube
3. Dermosphenotic participation in orbital margin

(Grande and Bemis, 1998; Xu et al., 2014; Xu and Shen, 2015; Sun et al., 2017; Ma and Xu, 2017; Xu and Ma, 2018; López-Arbarello and Sferco, 2018)

- - 1. Dermosphenotic reaches orbital margin
    2. Dermosphenotic does not reach orbital margin

1. Sphenotic with a relatively large exposed dermal component nearly reaching the orbital margin
2. Absent
3. Present
4. Dermosphenotic bone attachment to skull roof in adult-sized individuals

(Grande and Bemis, 1998; Brito and Alvardo-Ortega, 2013; Xu et al., 2014; Xu and Shen, 2015; Sun et al., 2017; Ma and Xu, 2017; Xu and Ma, 2018; López-Arbarello and Sferco, 2018)

1. Loosely attached on the skull roof or hinged to the side of skull roof
2. Firmly sutured into skull roof, forming part of it
3. Dermosphenotic/sphenotic association

(Grande, 2010; López-Arbarello and Sferco, 2018)

- - 1. Closely associated with each other (i.e. contacting or fused to each other)
    2. Not in contact with each other

1. Sclerotic ring ossification

(Grande and Bemis, 1998; Xu et al., 2014; Xu and Shen, 2015; Sun et al., 2017; Ma and Xu, 2017; Xu and Ma, 2018; Ebert, 2018; López-Arbarello and Sferco, 2018)

1. Present
2. Absent
3. Suborbital bones

(Grande and Bemis, 1998; Xu and Wu, 2012; Xu et al., 2014; Xu and Shen, 2015; Sun et al., 2017; Giles et al., 2017; Ma and Xu, 2017; Xu and Ma, 2018; Ebert, 2018; López-Arbarello and Sferco, 2018)

- - 1. Present
    2. Absent

1. Number of suborbital bones

(Xu and Gao, 2011; Xu et al., 2014; López-Arbarello and Wencker, 2016; Giles et al., 2017; López-Arbarello and Sferco, 2018)

- - 1. One
    2. Two or three
    3. Four or more

1. Arrangement of suborbital bones

(López-Arbarello, 2012; López-Arbarello and Sferco, 2018)

- - 1. One row
    2. Two or more rows

1. Suborbital bones extend anteriorly below the orbit

(López-Arbarello, 2012; López-Arbarello and Sferco, 2018)

- - - 1. Absent
      2. Present

**Palatoquadrate, hyoid and branchial arches**

1. Vomer in adults

(Brito and Alvardo-Ortega, 2013; Xu et al., 2014; Xu and Shen, 2015; Xu and Ma, 2016; Sun et al., 2017; Giles et al., 2017; Ma and Xu, 2017; Xu and Ma, 2018; López-Arbarello and Sferco, 2018)

- - 1. Paired
    2. Median

1. Autopalatine

(Grande, 2010; López-Arbarello, 2012; López-Arbarello and Wencker, 2016; Sun et al., 2017; López-Arbarello and Sferco, 2018)

- - 1. Present
    2. Absent

1. Endopterygoid/dermopalatine association.

(Grande, 2010; López-Arbarello and Sferco, 2018)

1. Endopterygoid sutured to dermopalatine anteriorly
2. Endopterygoid not in contact with any dermopalatine
3. Length of ectopterygoid relative to endopterygoid.

(Grande, 2010; Cavin et al., 2013; Deesri et al., 2016)

- 1. Twice the length of the endopterygoid or less
  2. More than twice the length of the endopterygoid

1. Quadrate/metapterygoid contact or close association.

(Grande, 2010; Deesri et al., 2016; López-Arbarello and Sferco, 2018)

1. Present
2. Absent
3. Laterally sliding articulation between metapterygoid and the parasphenoid-prootic process

(Grande, 2010; Cavin et al., 2013; Deesri et al., 2016; López-Arbarello and Sferco, 2018)

1. Absent
2. Present
3. Ectopterygoid participation in palatal surface area

(Grande, 2010, López-Arbarello, 2012; López-Arbarello and Sferco, 2018)

- - 1. Ectopterygoid form half or less of the palatal region
    2. Ectopterygoid forms the majority of the palatal region

1. Part of dorsal surface of ectopterygoid ornamented and forming part of skull roof

(Grande, 2010, López-Arbarello, 2012; Cavin et al., 2013; Deesri et al., 2016; López-Arbarello and Sferco, 2018)

- - 1. Absent
    2. Present

1. Urohyal

(Grande, 2010; López-Arbarello and Sferco, 2018)

1. Absent
2. Present
3. Basihyal tooth plate (‘tongue bone’) consisting of a mosatic of bony plates (‘entoglossals’). (Grande, 2010; Cavin et al., 2013; Deesri et al., 2016)
4. Absent
5. Present
6. Elongated posteroventral process of quadrate

(Arratia, 2013; Xu et al., 2014; Xu and Shen, 2015; Xu and Zhao, 2016)

1. Absent
2. Present
3. Quadrate

(Modified from Cavin, 2010; Cavin et al., 2013; Deesri et al., 2014, 2016; López-Arbarello and Sferco, 2018)

1. Not exposed (except condyle)
2. Partly or fully exposed
3. Quadrate almost fully covered by infraorbital(s)
   - 1. Absent
     2. Present
4. Symplectic

(Gardiner and Schaeffer 1989; Coates 1999; Hurley et al. 2007; Xu and Zhao, 2016; Giles et al., 2017; López-Arbarello and Sferco, 2018. The ossification that contacts the hyomandibula (and typically the quadrate), but does not articulate with the ceratohyal, is termed the symplectic. The ossification that forms an intermediary between the hyomandibula and ceratohyal is termed the interhyal. We follow Gardiner et al. (1996) in identifying the ‘interhyal’ of *Pteronisculus, Boreosomus,* and *Pycholepis* (Nielsen, 1942, 1949; Véran, 1981, 1988) as a posterior ceratohyal, and the ‘symplectic’ of these taxa as a interhyal.)

1. Absent
2. Present
3. Symplectic shape

(Grande, 2010; Cavin et al., 2013; Deesri et al., 2016; Giles et al., 2017; López-Arbarello and Sferco, 2018)

1. Slightly curved tube or splint;
2. Hourglass-shaped or hatchet-shaped;
3. L-shaped;
4. Membranous outgrowth of symplectic
5. Absent
6. Present
7. Position of the quadrate-mandibular articulation

(Modified from Grande, 2010; Xu et al., 2014; Xu and Shen, 2015; Sun et al., 2017; Ma and Xu, 2017; Xu and Ma, 2018; López-Arbarello and Sferco, 2018)

- - 1. Under the posterior border of the orbit or posterior to the orbit
    2. Under the orbit
    3. Under the anterior border of the orbit or anterior to the orbit

1. Quadratojugal

(Modified Grande, 2010; Cavin, 2010; Brito and Alvardo-Ortega, 2013; López-Arbarello, 2012; Cavin et al., 2013; Deesri et al., 2014, 2016; Xu and Zhao, 2016; Xu and Ma, 2016; Sun et al., 2017; Ma and Xu, 2017; Xu and Ma, 2018; López-Arbarello and Sferco, 2018)

- - 1. Present
    2. Absent

1. Shape of quadratojugal

(Gardiner et al., 1996; Hurley et al., 2007; López-Arbarello and Wencker, 2016)

- - 1. Plate-like
    2. Splint-like

1. Symplectic/quadrate contact

(Grande, 2010; Cavin et al., 2013; Xu et al., 2014; Xu and Shen, 2015; Deesri et al., 2016; Sun et al., 2017; Ma and Xu, 2017; Xu and Ma, 2018; Ebert, 2018; López-Arbarello and Sferco, 2018)

- - 1. Present
    2. Absent

1. Symplectic involvement in jaw joint

(Grande and Bemis, 1998; Grande 2010; Xu and Wu 2012; Brito and Alvardo-Ortega, 2013; Xu et al. 2014, 2015; Xu and Shen, 2015; Sun et al., 2017; Ma and Xu, 2017; Giles et al., 2017; Xu and Ma, 2018)

- - - 1. Absent
      2. Present

1. Preopercular process at the posterior margin of hyomandibula

(Arratia, 2013; López-Arbarello and Sferco, 2018)

1. Absent
2. Present
3. Number of hypobranchials

(Grande, 2010; Xu and Wu, 2012; Brito and Alvardo-Ortega, 2013; Xu et al., 2014; Xu and Shen, 2015; Xu and Ma, 2016; Xu and Zhao, 2016; Sun et al., 2017; Giles et al., 2017; López-Arbarello and Sferco, 2018)

1. Four
2. Three

**Jaws**

1. Olfactory nerve pierces premaxilla

(Grande, 2010; Xu and Wu, 2012; Cavin et al., 2013; Xu et al., 2014, 2015; Xu and Shen, 2015; Xu and Zhao, 2016; Deesri et al., 2016; Sun et al., 2017; Giles et al., 2017; Xu and Ma, 2018)

- - 1. Absent
    2. Present

1. Nasal process of premaxilla

(Gardiner and Schaeffer 1989; Gardiner et al. 1996; Gardiner et al. 2005; Cavin and Suteethorn 2006; Hurley et al. 2007; Grande 2010; López-Arbarello 2011; Xu and Wu 2012; Cavin et al., 2013; Xu et al. 2014; Xu and Shen, 2015; Xu and Zhao, 2016; Deesri et al., 2016; Giles et al., 2017; Xu and Ma, 2018)

- - 1. Absent
    2. Present

1. Length of the nasal process of premaxilla

(Grande, 2010; Xu and Wu, 2012; Xu et al., 2014; Xu and Shen, 2015; Sun et al., 2017; Xu and Ma, 2018; López-Arbarello and Sferco, 2018)

1. Relatively short, not sutured to frontals
2. Deep, tightly sutured to frontals
3. Nasal process of premaxilla forms much of the ornamented dermal roof in the snout region

(Grande, 2010; López-Arbarello, 2012; Xu and Ma, 2018)

- - 1. Absent
    2. Present

1. Supraorbital canal incorporated into premaxilla

(Grande, 2010; López-Arbarello, 2012; Cavin et al., 2013; Deesri et al., 2014, 2016; Xu and Ma, 2018)

- - 1. Absent
    2. Present

1. Premaxillary tooth row curves anteriorly at symphysis and laterally onto projecting horns as it nears frontal.

(Grande, 2010; Cavin et al., 2013; Deesri et al., 2014, 2016)

1. Absent
2. Present
3. Mobile maxilla in cheek

(Coates, 1999; Grande, 2010; Brito and Alvardo-Ortega, 2013; Xu et al., 2012; Xu et al., 2014; Xu and Shen, 2015; Sun et al., 2017; Giles et al., 2017)

1. Absent
2. Present
3. Maxilla extremely slender and rod-like

(Grande and Bemis, 1998; Xu et al., 2014; Xu and Shen, 2015; Sun et al., 2017; Ma and Xu, 2017; Xu and Ma, 2018)

1. Absent
2. Present
3. Peg-like anterior process of maxilla

(Grande, 2010; Xu and Wu 2012; Cavin et al., 2013; Xu et al. 2014; Deesri et al., 2016; Giles et al., 2017)

1. Absent
2. Present
3. Lateral line canal in maxilla

(Gardiner et al., 1996; Grande and Bemis, 1998; Brito and Alvardo-Ortega, 2013; Xu et al., 2014; Xu and Shen, 2015; Sun et al., 2017; Xu and Ma, 2018; López-Arbarello and Sferco, 2018)

1. Absent
2. Present
3. Posterior margin of maxilla

(Grande and Bemis, 1998; Xu and Wu 2012; Xu et al. 2014, 2015; Brito and Alvardo-Ortega, 2013; Arratia 2013; Xu and Zhao, 2016; Giles et al., 2017; Ma and Xu, 2017; Xu and Ma, 2018)

1. Convexly rounded or straight
2. Excavated
3. Coronoid process

(Gardiner and Schaeffer, 1989; Xu et al., 2014; Xu and Shen, 2015; Sun et al., 2017; Giles et al., 2017; Xu and Ma, 2018; López-Arbarello and Sferco, 2018)

1. Absent
2. Present
3. Composition of coronoid process
4. Formed by dentary and supra-angular
5. By dentary and angular
6. Mainly by dentary
7. Lacrimomaxillary bones

(Grande, 2010)

- - 1. Absent
    2. Present

1. Posterior end of maxilla relative to coronoid process

(López-Arbarello, 2012; López-Arbarello and Sferco, 2018)

- - 1. At the level of the coronoid process
    2. Anterior to the coronoid process
    3. Well posterior to the coronoid process

1. Posterior end of maxilla relative to orbit

(Modified from Gardiner et al., 1996; López-Arbarello and Sferco, 2018)

- - 1. Posterior to the orbit
    2. At the posterior border of the orbit
    3. Near the centre of the orbit
    4. At the anterior border of the orbit or anterior to the orbit

1. Oral margin of maxilla
   - 1. Slightly concave or straight
     2. Convex
2. Depth of maxilla

(Modified from López-Arbarello, 2012; López-Arbarello and Sferco, 2018)

- - 1. Shallow, < 0.5 of its length
    2. Deep, > 0.5 of its length

1. Suborbital/maxilla contact

(Xu et al., 2012, 2014, 2015; Xu and Ma, 2016)

- - 1. Present
    2. Absent

1. Supramaxilla

(Gardiner and Schaeffer, 1989; Grande and Bemis, 1998; Xu and Wu, 2012; Xu et al., 2014; Xu and Shen, 2015; Xu and Ma, 2016; Sun et al., 2017)

1. Absent
2. Present
3. Number of supramaxilla

(Grande and Bemis, 1998; Brito and Alvardo-Ortega, 2013; Xu et al., 2014; Xu and Shen, 2015; Sun et al., 2017; Ma and Xu, 2017; Xu and Ma, 2018)

1. one
2. two
3. Dorsal process of maxilla for single supramaxilla
   - 1. Present
     2. Absent
4. Teeth on maxilla

(López-Arbarello, 2012; Xu et al., 2014, 2015; Xu and Ma, 2016; Xu and Zhao, 2016; Giles et al., 2017)

- - 1. Present
    2. Absent

1. Plicidentine tooth structure

(Grande, 2010; López-Arbarello and Sferco, 2018)

- - 1. Absent
    2. Present

1. Coronoid bones

(Grande, 2010; Cavin et al., 2013; Deesri et al., 2016; López-Arbarello and Sferco, 2018)

1. Present as separate ossifications
2. Absent
3. Teeth on anterior coronoids

(Grande and Bemis, 1998; Xu et al., 2014; Xu and Shen, 2015; Sun et al., 2017; Ma and Xu, 2017; Xu and Ma, 2018; López-Arbarello and Sferco, 2018)

1. Conical or pointed pencil like
2. Styliform, with broadly rounded or flattened tips
3. Number of tooth rows on coronoids

(Grande and Bemis, 1998; Xu et al., 2014; Xu and Shen, 2015; Sun et al., 2017; Ma and Xu, 2017; Xu and Ma, 2018)

1. Two or more rows for at least part of one or more coronoids
2. One row
3. Arrangement of vomerine teeth

(Grande and Bemis, 1998; Xu et al., 2014; Xu and Shen, 2015; Sun et al., 2017; Ma and Xu, 2017; Xu and Ma, 2018)

1. Tooth patch with two to several rows of teeth
2. Tooth patch with only a single anterior marginal row, plus one or more teeth in a longitudinal series perpendicular to the anterior marginal row
3. Supra-angular

(Grande, 2010; Brito and Alvardo-Ortega, 2013; Cavin et al., 2013; Deesri et al., 2016; Xu et al., 2015; Xu and Ma, 2016, 2018; Ma and Xu, 2017; Xu and Ma, 2018; López-Arbarello and Sferco, 2018)

1. Present
2. Absent
3. ‘Leptolepid’ notch in ascending margin of dentary

(Arratia, 2013; Ma and Xu, 2017; Giles et al., 2017; Xu and Ma, 2018)

1. Absent
2. Present
3. Mobile premaxilla

(Arratia, 2013; Xu and Ma, 2016, 2018; Ma and Xu, 2017; Xu and Ma, 2018)

1. Absent
2. Present
3. Anterior end of first coronoid curves medially and expands broadly to a flat symphysis

(Grande, 2010)

1. Absent
2. Present
3. Mentomeckelian bone

(Grande, 2010; Cavin et al., 2013; Deesri et al., 2016)

1. Present
2. Absent
3. Type of dentary symphysis.

(Grande, 2010)

1. Symphysis occurs between the recurved anterior ends of right and left dentary
2. Symphysis occurs along medial surface of anterior right and left dentary with anterior ends pointing anterior
3. Prearticular.

(Grande, 2010; Cavin et al., 2013; Deesri et al., 2016; López-Arbarello and Sferco, 2018)

1. Present
2. Absent
3. Mandibular length as a percentage of head length.

(Grande, 2010; Cavin et al., 2013; Deesri et al., 2016)

1. 44% or more
2. Less than 43%
3. Supra-angular/angular contact
   - 1. Present
     2. Absent
4. Articular ossification of lower jaw

(Grande and Bemis, 1998; Xu et al., 2014; Xu and Shen, 2015; Sun et al., 2017; Ma and Xu, 2017; Xu and Ma, 2018; López-Arbarello and Sferco, 2018)

1. A single element, or two elements tightly sutured to each other
2. Two separate elements not in contact with each other
3. Teeth on dentary

(Xu et al. 2014; Xu and Ma, 2016; Giles et al., 2017; López-Arbarello and Sferco, 2018)

- - 1. Present
    2. Absent

1. Tooth organization on dentary

(Grande, 2010; Cavin et al., 2013; Deesri et al., 2016; López-Arbarello and Sferco, 2018)

1. Dentary teeth in a single row and all of similar size
2. In addition to a single row of similar sized teeth, there is a median row of much larger fangs
3. A pavement of small similar sized teeth not in rows
4. Well-developed posteroventral process of the dentary

(Cavin, 2010; López-Arbarello and Wencker, 2016; López-Arbarello and Sferco, 2018)

- - 1. Absent
    2. Present

1. Morphology of caps of the jaw teeth in adult-sized individuals

(Grande and Bemis, 1998; Xu et al., 2014; Xu and Shen, 2015; Sun et al., 2017; Ma and Xu, 2017; Xu and Ma, 2018)

1. Round in cross-section, not sharply carinate
2. Labiolingually compressed, sharply carinate (keeled)
3. Extent of teeth on dentary (excluding coronoid toothplates)

(Grande, 2010; López-Arbarello and Sferco, 2018)

- - 1. Tooth row extends over a third the length of dentary
    2. Tooth row is present on only the anterior one third or less of dentary

**Opercular series, branchiostegals and gular**

1. Shape of preopercle

(Grande and Bemis, 1998; Brito and Alvardo-Ortega, 2013; Xu et al., 2014; Xu and Shen, 2015; Sun et al., 2017; Ma and Xu, 2017; Xu and Ma, 2018; López-Arbarello and Sferco, 2018)

- - 1. Dorsally expanded, without anteroventral arm
    2. Crescent-shaped
    3. L-shaped
    4. Ovoid

1. Suborbtial between preopercle and dermopterotic
   - 1. Absent
     2. Present
2. Width of opercle

(Grande and Bemis, 1998; Xu et al., 2014; Xu and Shen, 2015; Sun et al., 2017; Ma and Xu, 2017; Xu and Ma, 2018)

1. Narrow, with width-to-height ratio of 0.56 to 1.06
2. Wide, with width-to-height ratio in range of 1.07 to 1.39
3. Exposure of dorsal limb of preopercle

(Grande, 2010; López-Arbarello and Sferco, 2018)

- - 1. Mostly exposed forming a significant part of the ornamented lateral surface of the skull anterior to the opercle
    2. Entirely covered or nearly entirely covered by other dermal bones in adults

1. Preopercle/dermopterotic contact

(Xu and Ma, 2016; López-Arbarello and Sferco, 2018)

1. Present
2. Absent
3. Posterior border of preopercle notched ventrally

(López-Arbarello, 2012; Giles et al., 2017)

- - 1. Absent
    2. Present

1. Subopercle with well-developed anterodorsal process

(López-Arbarello, 2012; Giles et al., 2017)

- - 1. Absent
    2. Present

1. Depth of ascending process of the subopercle

(López-Arbarello, 2012; López-Arbarello and Sferco, 2018)

- - 1. Less than or equal to half of the length of the opercle
    2. More than half of the length of the dorsal border of the bone

1. Interopercle

(Gardiner and Schaeffer 1989; Xu and Gao 2011; Xu et al. 2014, 2015. Gardiner and Schaeffer 1989; Olsen and McCune 1991; Gardiner et al. 1996; Gardiner et al. 2005; Cavin and Suteethorn 2006; Hurley et al. 2007; López-Arbarello 2012; Xu and Zhao, 2016; Giles et al., 2017)

- - 1. Absent
    2. Present

1. Position of anterior tip of the interopercle

(Olsen and McCune, 1991; López-Arbarello and Sferco, 2018)

- - 1. Close to the lower jaw
    2. Far away from the lower jaw

1. Lateral gulars

(Xu et al., 2014; Giles et al., 2017. The anterior most ‘fifth branchiostegal ray’ of the ‘*Perleidus*’ species from Madagascar is reinterpreted as the lateral gular because it has pit-lines.)

1. Present
2. Absent
3. Median gular.

(Grande and Bemis, 1998; Brito and Alvardo-Ortega, 2013; Xu et al., 2014; Xu and Shen, 2015; Sun et al., 2017; Ma and Xu, 2017; Xu and Ma, 2018)

- - 1. Present
    2. Absent

1. Number of branchiostegal rays

(Modified from Grande and Bemis, 1998; Xu et al., 2014; Xu and Shen, 2015; Sun et al., 2017; Ma and Xu, 2017; Xu and Ma, 2018; López-Arbarello and Sferco, 2018)

1. 10 to 20
2. 9 or fewer
3. 21 or more

**Vertebrate and caudal skeleton**

1. Solid, perichordally ossified, diplospondylous centra in adult-sized individuals.

(Grande and Bemis, 1998; Grande, 2010; Cavin et al., 2013; Xu et al., 2014; Xu and Shen, 2015; Deesri et al., 2016; Ma and Xu, 2017; Xu and Ma, 2018; Ebert, 2018)

1. Absent
2. Present
3. Anteriorly projecting spine-like processes on neural and/or haemal arches

(Grande and Bemis, 1998; Xu et al., 2014; Xu and Shen, 2015; Sun et al., 2017; Ma and Xu, 2017; Xu and Ma, 2018)

1. Absent
2. Present
3. Solid vertebral centra of adult-sized individuals

(Modified from Grande and Bemis, 1998; Xu et al., 2014; Xu and Shen, 2015; Sun et al., 2017; Ma and Xu, 2017; Xu and Ma, 2018; Ebert, 2018)

1. Absent
2. Present
3. Surface of solid vertebral centra

(Modified from Grande and Bemis, 1998; Ma and Xu, 2017; Xu and Ma, 2018)

1. Smooth
2. Two or more lateral fossae on each side of most centra
3. Number of supraneurals

(Grande and Bemis, 1998; Xu et al., 2014; Xu and Shen, 2015; Sun et al., 2017; Ma and Xu, 2017; Xu and Ma, 2018)

1. 13 or more
2. 5 to 11
3. Opistocoelus vertebrae
   - 1. Absent
     2. Present
4. Hypural-ural centra fusion in adult-sized individuals

(Grande and Bemis, 1998; Xu et al., 2014; Xu and Shen, 2015; Sun et al., 2017; Ma and Xu, 2017; Xu and Ma, 2018)

1. All hypurals autogenous (separate) from the ural centra
2. All but first hypural fused to corresponding centra
3. Large parapophyses fused to most of the abdominal centra

(Grande and Bemis, 1998; Xu et al., 2014; Xu and Shen, 2015; Sun et al., 2017; Ma and Xu, 2017; Xu and Ma, 2018; López-Arbarello and Sferco, 2018)

1. Absent
2. Present
3. Number of preural vertebral centra

(Grande and Bemis, 1998; Xu et al., 2014; Xu and Shen, 2015; Sun et al., 2017; Ma and Xu, 2017; Xu and Ma, 2018)

1. 40 to 73
2. 75 to 82
3. One-to-one arrangement of hypurals and caudal fin rays

(Grande and Bemis, 1998; Brito and Alvardo-Ortega, 2013; Xu et al., 2014; Xu and Shen, 2015; Sun et al., 2017; Ma and Xu, 2017; Xu and Ma, 2018; López-Arbarello and Sferco, 2018)

1. Last few hypurals each articulate with bases of several caudal fin rays
2. Each hypural normally bears a single caudal ray
3. Number of ossified ural neural arches in adult-sized individuals

(Grande and Bemis, 1998; Brito and Alvardo-Ortega, 2013; Xu et al., 2014; Xu and Shen, 2015; Sun et al., 2017; Ma and Xu, 2017; Xu and Ma, 2018; López-Arbarello and Sferco, 2018)

1. Normally four or more
2. Normally 2 or fewer
3. Numerous paired, block-like ural neural arch ossifications

(Grande and Bemis, 1998; Xu et al., 2014; Xu and Shen, 2015; Sun et al., 2017; Ma and Xu, 2017; Xu and Ma, 2018)

1. Absent
2. Present
3. Long epineural intermuscular bones

(Grande, 2010; Cavin et al., 2013; Deesri et al., 2016; López-Arbarello and Sferco, 2018)

1. Absent
2. Present
3. Articulation of proximal end of pleural ribs with vertebral parapophyses

(Modified from Grande, 2010)

1. Posterior to parapophyses;
2. Anteroventral to parapophyses;
3. Shape of haemal spines

(Grande and Bemis, 1998; Xu et al., 2014; Xu and Shen, 2015; Sun et al., 2017; Ma and Xu, 2017; Xu and Ma, 2018; López-Arbarello and Sferco, 2018)

1. Spine-like or rod-like
2. Broadly spatulate in the transverse plane
3. Orientation of preural haemal and neural spines near caudal peduncle

(Grande and Bemis, 1998; Xu et al., 2014; Xu and Shen, 2015; Sun et al., 2017; Ma and Xu, 2017; Xu and Ma, 2018)

1. Positioned at about 25º to 45 º from the horizontal
2. Strongly inclined to nearly horizontal
3. Uroneurals

(Modified from Arratia, 2013; Xu et al., 2014; Xu and Shen, 2015; Xu and Ma, 2016, 2018; Sun et al., 2017; Ma and Xu, 2017; López-Arbarello and Sferco, 2018)

1. Absent
2. Present, both preural and ural neural arches modified as uroneurals
3. Present, only ural neural arches modified as uroneurals
4. A gap between hypurals 2 and 3

(Arratia, 2013; Xu et al., 2014; Xu and Shen, 2015; Sun et al., 2017; ; Ma and Xu, 2017; Xu and Ma, 2018; López-Arbarello and Sferco, 2018)

1. Absent
2. Present

**Girdles**

1. Anteroventral process of posttemporal bone

(Grande, 2010; Cavin et al., 2013; Deesri et al., 2016)

- - 1. Absent
    2. Weakly developed
    3. Well developed as a ventral rod-like process

1. Posttemporal penetration by lateral line canal

(Grande, 2010; López-Arbarello, 2012; Cavin et al., 2013; Deesri et al., 2014; López-Arbarello and Sferco, 2018)

- - 1. Present
    2. Absent

1. Width of the posttemporal
   - 1. Broad, reaching the midline
     2. Relatively narrow, not reaching the midline (separated by scales)
     3. Much narrow, narrower than the dermopterotic
2. Posterodorsal margin of the supracleithrum peculiarly ornamented
   - 1. Absent
     2. Present
3. Supracleithrum with a concave articular facet for articulation with the posttemporal

(Grande, 2010; López-Arbarello and Sferco, 2018)

1. Absent
2. Present
3. Length of anterior arm of cleithrum relative to depth of its dorsal arm

(Xu and Ma, 2018)

1. Anterior arm shorter than or nearly equal to dorsal arm
2. Anterior arm notably longer than dorsal arm
3. Substantial scapulocoracoid ossification in adult-sized individuals

(Grande and Bemis, 1998; Xu et al., 2014; Xu and Shen, 2015; Sun et al., 2017; Ma and Xu, 2017; Xu and Ma, 2018)

1. One or more elements present in the shoulder girdle
2. Absent
3. Medial processes of suprascapula.

(Grande, 2010; Cavin et al., 2013; Deesri et al., 2016)

1. Absent
2. Present
3. Clavicle anterior to cleithrum

(Grande, 2010; Cavin et al., 2013; Deesri et al., 2016; Xu and Ma, 2018; López-Arbarello and Sferco, 2018)

1. Present
2. Absent
3. Anterior and posterior ‘clavicle elements’

(Grande, 2010; Cavin et al., 2013; Deesri et al., 2016; Xu and Ma, 2018; López-Arbarello and Sferco, 2018)

1. Absent
2. Present
3. Medial wing on cleithrum.

(Cavin and Suteethorn, 2006; Grande, 2010; Cavin et al., 2013; Cavin et al., 2013; Deesri et al., 2016; Giles et al., 2017)

1. Present
2. Absent
3. Presupracleithrum

(Gardiner et al., 2005; Xu et al., 2014; Giles et al., 2017)

1. Present
2. Absent
3. Pectoral propterygium fused with first ray

(Arratia, 2013)

1. Absent
2. Present

**Fins**

1. Shape of posterior margin of caudal fin

(Grande and Bemis, 1998; Brito and Alvardo-Ortega, 2013; Xu et al., 2014; Xu and Shen, 2015; Sun et al., 2017; Ma and Xu, 2017; Xu and Ma, 2018)

1. Forked
2. Convexly rounded or nearly vertical
3. Fringing fulcra on pectoral fins
   - 1. Present
     2. Absent
4. Fringing fulcra on pelvic fins
   - 1. Present
     2. Absent
5. Fringing fulcra on median fins

(Grande and Bemis, 1998; Xu et al., 2014; Xu and Shen, 2015; Sun et al., 2017; Ma and Xu, 2017; Xu and Ma, 2018)

1. Present
2. Absent
3. Number of dorsal fin(s)

(Ebert, 2018)

- - 1. Single
    2. Two

1. Shape of (first) dorsal fin

(Gardiner and Schaeffer, 1989)

- - 1. Triangular
    2. Bow-shaped

1. Origin of (first) dorsal fin

(López-Arbarello, 2012)

- - 1. Posterior to the origins of pelvic fins
    2. Opposite or anterior to the origins of pelvic fins

1. Number of dorsal fin rays

(López-Arbarello, 2012; Deesri et al., 2016)

- - 1. 20 or more
    2. Less than 20

1. Dorsal and anal fin rays

(Modified from Gardiner et al., 1996; Xu and Gao, 2011; Xu and Wu, 2012; Xu et al., 2012, 2014, 2015)

1. rays more numerous than radials
2. rays and radials largely equal in number
3. Number of principal caudal fin rays below the lateral line in adults

(López-Arbarello, 2012; López-Arbarello and Wencker, 2016)

- - 1. Nine or more
    2. Eight or seven
    3. Six or less

1. Predorsal length
   - 1. 70% or less of SL
     2. 75% or more of SL
2. Enlarged dorsal scute (corresponding to more than one vertical row of scale) preceding caudal fin

(Arratia, 2013)

1. Absent
2. Present
3. Number of epaxial procurrent caudal fin rays

(Grande and Bemis, 1998; Xu et al., 2014; Xu and Shen, 2015; Sun et al., 2017; Ma and Xu, 2017; Xu and Ma, 2018)

1. 0 to 11
2. 12 to 15
3. Lateral line ossicles extending onto caudal fin

(Gardiner et al., 1996; Xu et al., 2014; Xu and Shen, 2015; Sun et al., 2017; ; Ma and Xu, 2017; Xu and Ma, 2018; López-Arbarello and Sferco, 2018)

1. Absent
2. Present

**Body shape and scales**

1. An apparent dorsal hump between head and dorsal fin.

(Xu and Wu, 2012).

1. Absent
2. Present
3. Scales

(Alvarado-Ortega and Espinosa-Arrubarrena, 2008; Brito and Alvardo-Ortega, 2013; Xu et al., 2012; Xu et al., 2014; Xu and Shen, 2015; Sun et al., 2017; Ma and Xu, 2017; Xu and Ma, 2018; Ebert, 2018)

1. Rhomboid
2. Amioid-type, subrectangular to elongate oval
3. Elasmoid of cycloid type
4. Dorsal peg of rhomboid scale

(Modified from López-Arbarello, 2012; Deesri et al., 2016)

- - 1. Present
    2. Absent

1. Anteroventral articular process of rhomboid scale
   - 1. Absent
     2. Present
2. Spines on dorsal ridge scales anterior to dorsal fin

(Olsen and McCune, 1991; Grande, 2010; Xu and Wu, 2012; López-Arbarello, 2012)

- - 1. Absent
    2. Present

1. Flank scales with large prominent posteriorly pointing spines

(Grande, 2010; López-Arbarello, 2012; Cavin et al., 2013; Deesri et al., 2016)

- - 1. Absent
    2. Present

1. Complete row of elongated scales between last lateral line scale and uppermost caudal fin ray

(López-Arbarello, 2012; López-Arbarello and Wencker, 2016; López-Arbarello and Sferco, 2018)

- - 1. Absent
    2. present

1. Urodermals in the caudal

(Grande and Bemis, 1998; Xu et al., 2014; Xu and Shen, 2015; Sun et al., 2017; Ma and Xu, 2017; Xu and Ma, 2018)

1. Present
2. Absent

Part C. Data matrix of taxa and characters

*Pteronisculus_stensioi*

0---0----00-00-0000?000000000100000000000---000---0000000000000000100??000000000000--000?00000-000000000-0-00000--0000000000000000000000000000-0-000000-00?0?0000-000000000000000000000000000000000000000

*Teffichthys_madagascariensis*

1000?00??0100000000?000000000000000000001000000---10000000000000?0000??00???0??000???01-?00?00-000000000-0-00010--000????000?0000?000000000000-0-0010?0-?0?0?0????????000000???000?0000000110000000000000

*Watsonulus_eugnathoides*

1?00000?002000?10000020000000000111000011111000---101001100000000021000000000??000110000010?01100010101100000011000000000000000000001003100101010100000-000000?00?00002000?000000000000000110000000000000

*Amia_calva*

10010110002000110010020110-00000111101010---010---0010111000001011---000000000001011101-0100111000101011000100-10000000000000000010000010000010101001?101011111001000020000110111101111011010000101--0001

*Caturus_furcatus*

1?01011?002000110000020000000000111001011011000---0000011000001000100000000000001011001-010011100011101100010011010000?10000000000001101000001010102000-000000010?11002000?100111000000001110000001--00?0

*Quetzalichthys*

???????????????1????02010010000011100?011?11110---01110110010110?0100???????0??0001??01-010??11000101111?00000110100000?000??0?00?0000010000010101000?110??00000??0000?000100?1????0?00000010000001--00?0

*Ionoscopus_cyprinoides*

1201000100210011001002010000000011100?0110110?0---01100110010110001000?0?00000001011001-010??1100010111100000011010000010000?00000000001100101010100001100000000?10000200010001???00000000110000001--0000

*Ophiopsiella_procera*

???????????100?1????020000101000111000011010110---00110111110110001000????0?0??0001?001-01???110001011110001001100000?0?000??0?00?0010010000010101001?100?0000?0??0000?000?00?1???00000001010000100000000

*Asialepidotus*

????????002?00?1???0020100100200111000011111100---00000110010110001000?000?00??010110001010001100010111100010011000000000000?0000?001001000001010100??0-?0?????????????00010??110000000000110000000000000

*Solnhofenamia*

??????1?002?00?1?01?020000000000111000011011000---00000110000010000000?0000000001011001-010??1100010101100010011010000000000000000000001010001010100111000000110010000200001001?1101111011010001001--0000

*Elops_hawaiensis*

10010100002010000001030000100000-31100001211000---1010011000000011---100000001011010001-000100-000101001202010-11-001--0101-1010-0021002000000-1011200110001000010002120000000101110111001110010002--0000

*Leptolepis_coryphaenoides*

10010100002011000001030000100000-31000001100000---10000110000000?00001?0000001011010011-001?00-000101001102210111-001--?111-1001-0001002101100-101000010?0000000100?2120000000101110111001110010002--0001

*Dorsetichthys*

10010000002011000001010000100000020000001100000---10000110000000000001?0000001011010001-000?00-000101001002110111-00000?0110100000001002100111010100000-?0000000100011?0000000??1?10000000110010000000000

*Ichthyokentema*

11010100002010000001010100000000111000001011000---1000011000000000000100?0000??11010001-000?00-0001010011002101100000000111?10?0-?001002000001010100??10?0????????????2000?0??1?11?0000000110010000000000

*Pholidoctenus*

???????????????0???0010---1--00011000-001100000---1000011000000?00100??0?0??0???00???01-?0??00-0001010(01)1?02210111-00????011??0?00?001002100101010100??0-?0??????0?00?1?000?0????1000000000110010000000010

*Pliodetes_nigerienesis*

????????????????????02010011110011100101111000101100100??100000??02000?????00???00???10???0?111110101001?0120?10--10???0100??0?11?0?0?1200000100-11???1??000??????????0010?0??1????000000011?00?0000001?0

*Tlayuamichin_itztli*

????????0??????1???0020100111100111000?1100000120000100331000000?01000???0?00???10???2?100???110001010010003011101100110000??0?100001001100101110111?????0????????????001010??1??100000000111000000011000

*Macrosemius_rostratus*

?11-110?0?2-??000000020100?11011111?0101????0012-00010033100000011---0?00000000010?0?201000?010000101001?00300-0--00010?00001001?0000002000111011111??0-?0?1????0??????01010?01?010111101101100000000-0?0

*Notagogus_denticulatus*

???????????????????0020?001110111?1001011000001?-00010033100?00011---??????00???10???201?00??11000101001?00300-0--00????0000?0?1??000002000111011111?????0??????0??????01010??1??1101001010110000000000?0

*Propterus_elongatus*

????1??????????0???00201001110111110010110000012000010033100000011---0?????00???101?0201?00??11000101001?00300-0--00????0000?0?1??000002000011011111??0-10??0??????0?0?01010??11?100100101011000000000000

*Kyphosichthys_grandei*

????????????????????0201000110101110000111000010001010022100000000100???????0??001???001?0???1100010100100020011000000??000??0?10?0010011001010101010?0-???0???????????01010?????100000000110000010001010

*Macrosemimimus_fegerti*

????0???1??????1????0201001110101110000110000012000010033100000000100???0??00???10???201?0??1110001010010003001101100100000???010?001001100101110111???????????????????01110??1??1000000001110000000?1000

*Semionotus_elegans*

?11-?1??102-00?100?0020(01)001110001110000110100010110010033000000??000000??000000010100101000011100010100100030011010000000000100100001001000001010111000-000000000?0000201010?0110100000000111000000001000

*Lepisosteus_osseus*

011-1110102-0000101002010111120011101111111000122110100331000001102100111111101001120201100011111010000101----10---10001000011001001000201100100-11100110101010001000011200001110101000000112100000000000

*Atractosteus_spatula*

0(01)1-1110102-0000101002010011120011101111111000122110100331000001102100111111101001120201100011111010000101----10---10001000110001001000201100110-11100110101010001000011200001110101000000112100000100000

*Obaichthys_decoratus*

?1??1110102?000111101211001112001110111110100012111010033100?00?102100??0111001010???201?000?111111010010013001???00000?00001001100000120100010111110011?101?100010?00??2000000001010000001121000000001?0

*Dentilepisosteus_laevis*

?10?11101020000110101211001111001110111110100012211010033100?00?102100110111001010???201?00?111111101001001300110100000?00001001?00000120100010111110011?10101000?00002020?0001?01?1000000112100000000100

*Masillosteus_janeae*

??1-?11?102-00?0?0100201001111001110111111100012211010033100(01)00110210????1?10??001???201?00?1111?0?0?00101----10---1010?000010011000001200100100-1110011?10101000?0000112000??1?01?1000000112100000000000

*Cuneatus_wileyi*

0????110102?00???0?00201001112001110?1111(01)100012211010033100000110210??111110??001???201?00?111110?0?00101----10---1000?000010011000000200100110-1110011?10101000?00001120000?1?01?10000001121000000?0000

*Araripelepidotes_temnurus*

11??0100022?00?000000201100111001?1001?111?00010?010100??100000??0100??????00??0011?01??1??????0??101000-0120010--1??????00??0?11?1-0?1200000101111???0-?0?????????????01000??1???00000000111000000010100

*Fuyuanichthys_wangi*

?????????2?????????0020100-11010111100010---0010-01010022100000??0110??????00???01???101?0???1100010100100030010--10?0??000????10?001011100101010101??0-?0?????????????010?0?????1?000000011(01)000000001010

*Lepidotes_semiserratus*

110?0??0???0??010010020100111000111000-1111000112100100331000000?0201??????00??00010010100???110?0101001?003001101000???000??0?1??001001000001010111??0-?0?????????????0?010??1??1?0000000?1000?0000100?0

*Lepidotes_gigas*

???????????-???1???00201001110001110?0?1111000112100100331000000?0201?0?0??00???00???101?0???11000101001000300110100?0??000??0?10?001001000001010111??0-?0????????????001010??1??1000?000011000?000010000

*Thaiichthys_buddhabutrensis*

1(01)0?11??12200001?00002010011120011100111111000112110100331000001?01000?00??000?001100101100?111000101001?0130010--00???0000??0010?00100200000101?11??00-00??0?????????0010?0??1?0??000000011000?0000110?0

*Ticinolepis_longaeva*

????????2??000?????0020000000000111001011(01)0000100000100220000000?021100?????0??000100001000?01100010100100020011000000000000??010?001001000001010111??0-?0?????????????00010????00?0000000110000000000000

*Semiolepis_brembanus*

???????????????????00201001112001?100??1100000110010100331000000?0000???0??00???10???1????0??11000101001?00300110?00010??00??0?1??00100100000101111??????0?????????????01010???????0000000110000000001000

*Isanichthys_palustris*

????????1??????0????0201001011001?1000??1110001?210010033100100??0201???????????00???1????????????101001000000110100????000??0?10?0?1001000001010111??0-?0?????????????010?????????0??0000?1?000000??00?0

Part D. Supplementary figures


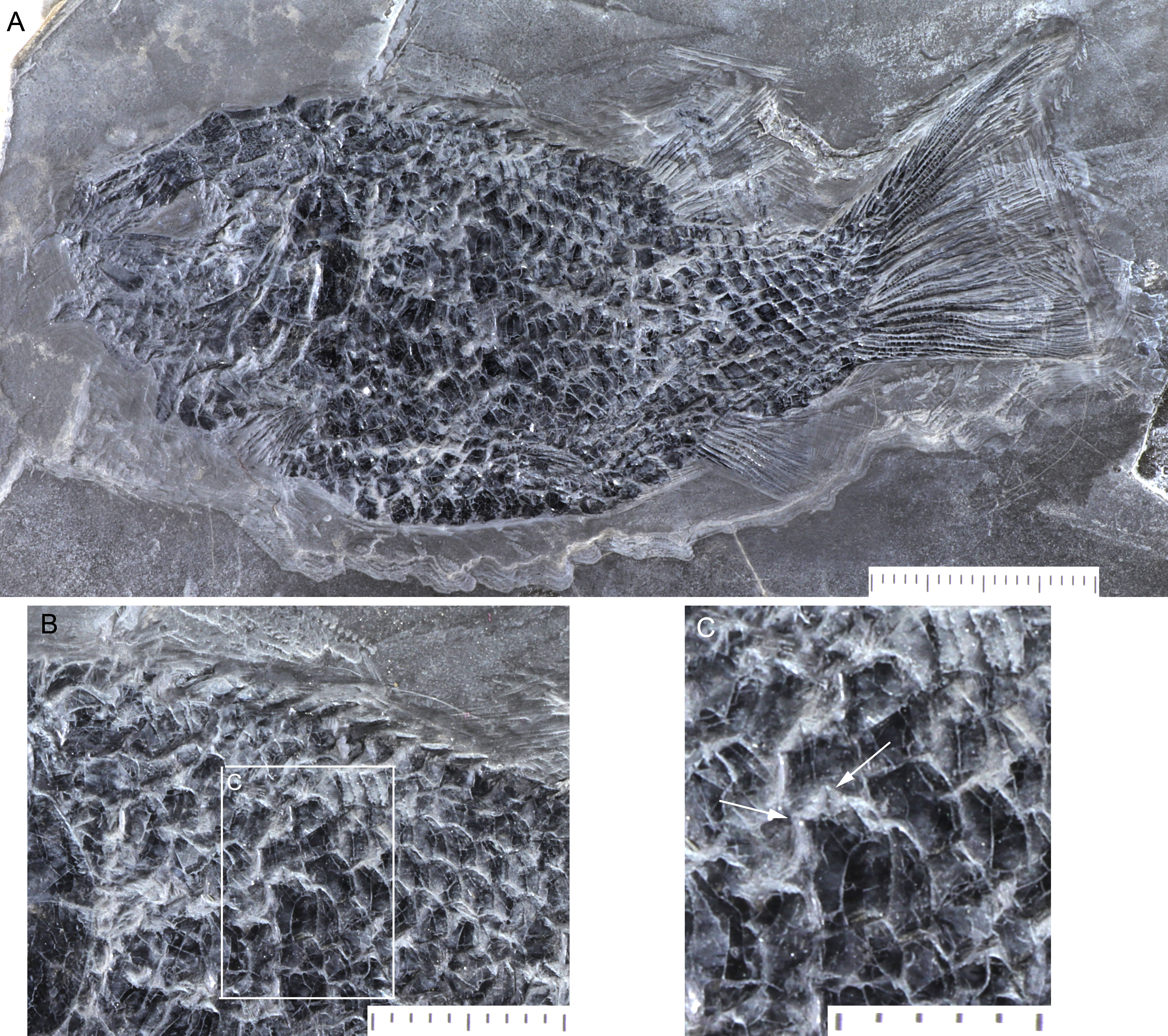


**Figure 1S.** ***Fuyuanichthys wangi* gen. et sp. nov.** **, IVPP V19992.** Complete skeleton (A) and close-up of scales in the anterior flank region (B, C) with arrows indicating the dorsal peg and anterodorsal extension of the scale.


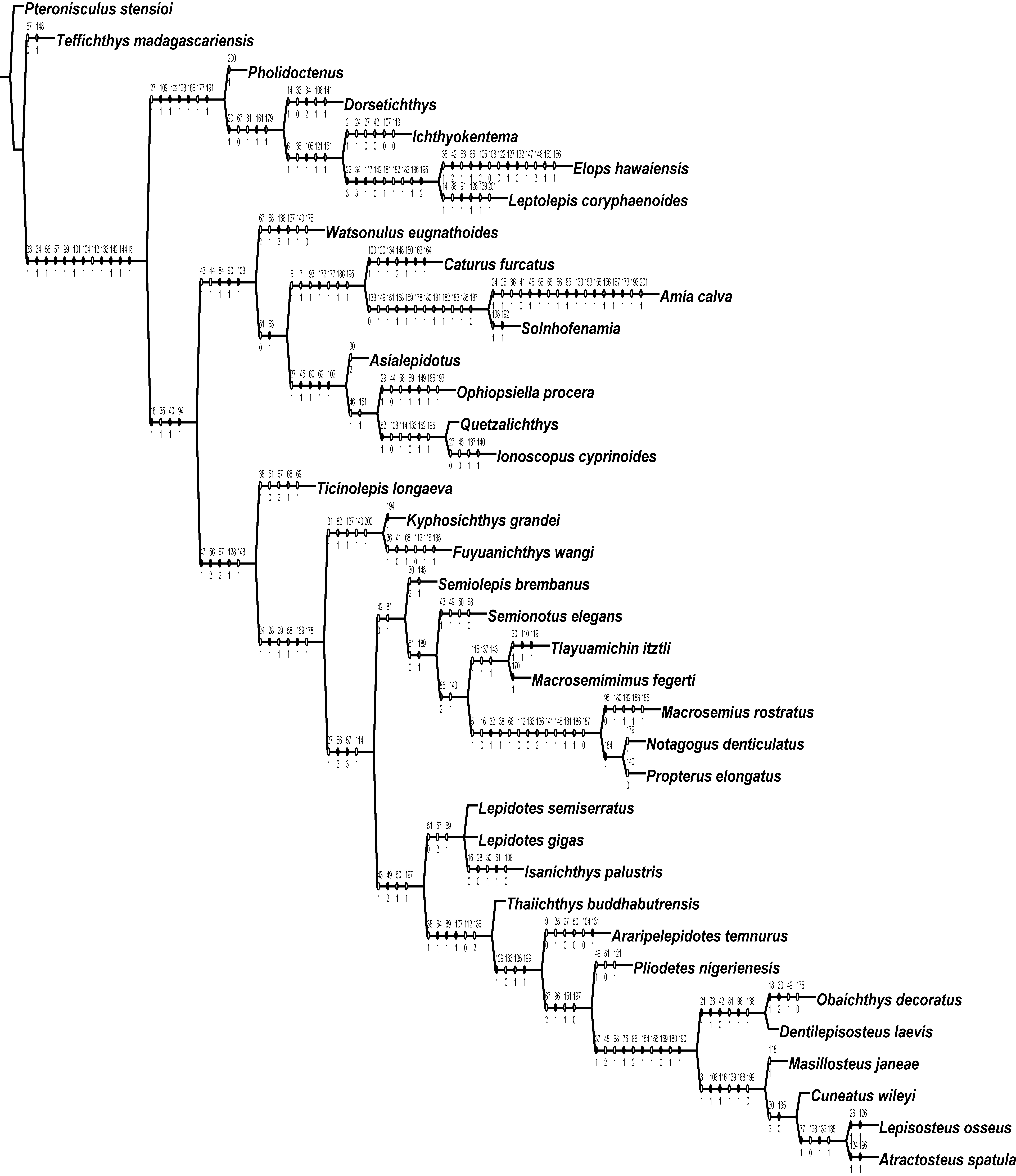


**Figure 2S.** Strict consensus of three most parsimonious trees (tree length = 478 steps, consistency index = 0.5021, retention index = 0.7503), illustrating the phylogenetic relationships of *Fuyuanichthys* within the Neopterygii. Character changes indicated with solid circles are unique.

Part E. Literature cited

Alvarado-Ortega, J., and L. Espinosa-Arrubarrena. 2008. A new genus of ionoscopiform fish (Halecomorphi) from the Lower Cretaceous (Albian) lithographic limestones of the Tlayúa quarry, Puebla, México. Journal of Paleontology 82:163–175.

Arratia, G. 2013. Morphology, taxonomy, and phylogeny of Triassic pholidophorid fishes (Actinopterygii, Teleostei). Society of Vertebrate Paleontology Memoir (Supplement to Journal of Vertebrate Paleontology) 13:1–138.

Brito, P. M., and J. Alvarado-Ortega. 2013. *Cipactlichthys scutatus*, gen. nov., sp. nov. a new halecomorph (Neopterygii, Holostei) from the Lower Cretaceous Tlayúa Formation of Mexico. PLoS ONE 8:e73551.

Cavin, L. 2010. Diversity of Mesozoic semionotiform fishes and the origin of gars (Lepisosteidae). Naturwissenschaften 97:1035–1040.

Cavin, L., and V. Suteethorn. 2006. A new Semionotiform (Actinopterygii, Neopterygii) from Upper Jurassic–Lower Cretaceous deposits of North-East Thailand, with comments on the relationships of Semionotiforms. Palaeontology 49:339–353.

Cavin, L., U. Deesri, and V. Suteethorn. 2013. Osteology and relationships of *Thaiichthys* nov. gen.: a Ginglymodi from the Late Jurassic–Early Cretaceous of Thailand. Palaeontology 56:183–208.

Coates, M. I. 1999. Endocranial preservation of a Carboniferous actinopterygian from Lancashire, UK, and the interrelationships of primitive actinopterygians. Philosophical Transactions of the Royal Society of London B 354:435–462.

Deesri, U., K. Lauprasert, V. Suteethorn, K. Wongko, and L. Cavin. 2014. A new species of the ginglymodian fish *Isanichthys* from the Late Jurassic Phu Kradung Formation, northeastern Thailand. Acta Palaeontologica Polonica 59:313–331.

Deesri, U., P. Jintasakul, and L. Cavin. 2016. A new Ginglymodi (Actinopterygii, Holostei) from the Late Jurassic–Early Cretaceous of Thailand, with comments on the early diversification of Lepisosteiformes in Southeast Asia. Journal of Vertebrate Paleontology 36: e1225747.

Ebert, M. 2018.*Cerinichthys koelblae*, gen. et sp. nov., from the Upper Jurassic of Cerin, France, and its phylogenetic setting, leading to a reassessment of the phylogenetic relationships of Halecomorphi (Actinopterygii). Journal of Vertebrate Paleontology 38:e1420071.

Gardiner, B. G., and B. Schaeffer. 1989. Interrelationships of lower actinopterygian fishes. Zoological Journal of the Linnean Society 97:135–187.

Gardiner, B. G., J. G. Maisey, and D. T. J. Littlewood. 1996. Interrelationships of basal neopterygians; pp. 117–146 in M. L. J. Stiassney, L. R. Parenti, and G. D. Johnson (eds.), Interrelationships of fishes. Academic Press, San Diego.

Giles, S., G.-H. Xu, T. J. Near, and M. Friedman. 2017. Early members of ‘living fossil’ lineage imply later origin of modern ray-finned fishes. Nature 549:265–268.

Grande, L. 2010. An empirical synthetic pattern study of gars (Lepisosteiformes) and closely related species, based mostly on skeletal anatomy. The resurrection of Holostei. Copeia 10 (Supplement):1–871.

Grande, L., and W. E. Bemis. 1998. A comprehensive phylogenetic study of amiid fishes (Amiidae) based on comparative skeletal anatomy: An empirical search for interconnected patterns of natural history. Society of Vertebrate Paleontology Memoir (supplement to Journal of Vertebrate Paleontology) 4:1–690.

López-Arbarello, A. 2102. Phylogenetic interrelationships of ginglymodian fishes (Actinopterygii: Neopterygii). PLoS ONE 7:e39370.

López-Arbarello, A., and E. Sferco. 2018. Neopterygian phylogeny: the merger assay. Royal Society Open Science 5:172337.

López-Arbarello, A., and J. Alvarado-Ortega. 2011. New semionotiform (Neopterygii) from the Tlayúa Quarry (Early Cretaceous, Albian), Mexico. Zootaxa 2749:1–24.

López-Arbarello, A., and L. C. M. Wencker. 2016. New callipurbeckiid genus (Ginglymodi: Semionotiformes) from the Tithonian (Late Jurassic) of Canjuers, France. Paläontologische Zeitschrift 90:543–560.

López-Arbarello, A., R. Stockar, and T. Bürgin. 2014. Phylogenetic relationships of the Triassic *Archaeosemionotus* Deecke (Halecomorphi, Ionoscopiformes) from the ‘Perledo Fauna’. PLoS ONE 9:e108665.

Ma, X.-Y., and G.-H. Xu. 2017. A new ionoscopiform fish (Holostei: Halecomorphi) from the Middle Triassic (Anisian) of Yunnan, China. Vertebrata PalAsiatica 55:92–106.

Olsen, P. E. 1984. The skull and pectoral girdle of the parasemionotid fish *Watsonulus eugnathoides* from the Early Triassic Sakamena Group of Madagascar, with comments on the relationship of the holostean fishes. Journal of Vertebrate Paleontology 4:481–499.

Olsen, P. E., and A. R. McCune. 1991. Morphology of the *Semionotus elegans* species group from the Early Jurassic part of the Newark supergroup of eastern North America with comments on the family Semionotidae (Neopterygii). Journal of Vertebrate Paleontology 11:269–292.

Sun, Z.-Y., A. Tintori, Y.-Z. Xu, L. Cristina, P.-G. Ni, and D.-Y. Jiang. 2017. A new non-parasemionotiform order of the Halecomorphi (Neopterygii, Actinopterygii) from the Middle Triassic of Tethys. Journal of Systematic Palaeontology 15:223–240.

Sun, Z.-Y., and P.-G. Ni. 2018. Revision of *Kyphosichthys grandei* Xu and Wu, 2012 from the Middle Triassic of Yunnan Province, South China: implications for phylogenetic interrelationships of ginglymodian fishes Journal of Systematic Palaeontology 16:67–85.

Xu, G.-H., and C.-C. Shen. 2015. *Panxianichthys imparilis* gen. et sp. nov., a new ionoscopiform (Halecomorphi) from the Middle Triassic of Guizhou, China. Vertebrata PalAsiatica 53:1–15.

Xu, G.-H., and F.-X. Wu. 2012. A deep-bodied ginglymodian fish from the Middle Triassic of eastern Yunnan Province, China, and the phylogeny of lower neopterygians. Chinese Science Bulletin 57:111−118.

Xu, G.-H., and K.-Q. Gao. 2011. A new scanilepiform from the Lower Triassic of northern Gansu Province, China, and phylogenetic relationships of non-teleostean Actinopterygii. Zoological Journal of the Linnean Society 161:595–612.

Xu, G.-H., and L.-J. Zhao. 2016. A Middle Triassic stem-neopterygian fish from China shows remarkable secondary sexual characteristics. Science Bulletin 61:338–344.

Xu, G.-H., and X.-Y. Ma. 2018. Redescription and phylogenetic reassessment of *Asialepidotus shingyiensis* (Holostei: Halecomorphi) from the Middle Triassic (Ladinian) of China. Zoological Journal of the Linnean Society. Doi: 10.1093/zoolinnean/zlx105/4827721.

Xu, G.-H., K.-Q. Gao, and M. I. Coates. 2015. Taxonomic revision of *Plesiofuro mingshuica* from the Lower Triassic of northern Gansu, China, and the relationships of early neopterygian clades. Journal of Vertebrate Paleontology 35:e1001515.

Xu, G.-H., L.-J. Zhao, and M. I. Coates. 2014. The oldest ionoscopiform from China sheds new light on the early evolution of halecomorph fishes. Biology Letters 10:20140204.

Xu G-H, Gao K-Q, Finarelli JA. 2014. A revision of the Middle Triassic scanilepiform fish *Fukangichthys longidorsalis*from Xinjiang, China, with comments on the phylogeny of the Actinopteri. Journal of Vertebrate Paleontology 34: 747–759.

Xu, G.-H., L.-J. Zhao, K.-Q. Gao, and F.-X. Wu. 2012. A new stem-neopterygian fish from the Middle Triassic of China shows the earliest over-water gliding strategy of the vertebrates. Proceedings of the Royal Society, Series B 280:20122261.
